# Iatrogenic coronal-sagittal coupling driven by a 12.4° rotational mismatch in manual total knee arthroplasty and precise decoupling with robotic assistance: a radiographic retrospective cohort study

**DOI:** 10.1186/s42836-026-00398-3

**Published:** 2026-06-03

**Authors:** Hongxu Li, Qihan Ma, Haoyang Liu, Hangyu Ping, Mengran Shen, Debo Yue, Bailiang Wang, Jinhui Ma

**Affiliations:** 1 Department of Orthopaedic Surgery, Peking University China-Japan Friendship School of Clinical Medicine, Beijing, 100029 China; 2 Department of Orthopaedic Surgery, Center for Osteonecrosis and Joint Preserving & Reconstruction, China-Japan Friendship Hospital, Beijing, 100029 China; 3 China-Japan Friendship Hospital (Institute of Clinical Medical Sciences), Chinese Academy of Medical Sciences & Peking Union Medical College, Beijing, 100730 China; 4 Jiangsu Province (Suqian) Hospital, Suqian, Jiangsu 223800 China

**Keywords:** Total knee arthroplasty, Robotic-assisted surgery, Coronal-sagittal coupling, Rotational alignment, Combined flexion

## Abstract

**Background:**

Achieving precise reconstruction of both the coronal and sagittal planes is pivotal in total knee arthroplasty (TKA). However, conventional manual TKA (M-TKA) may induce an unintended “coronal-sagittal coupling” effect, where adjustments in one plane inadvertently alter the outcome in the other. This study aimed to quantify this iatrogenic coupling effect in M-TKA and to evaluate the potential benefits of robotic-assisted TKA (R-TKA) in decoupling these two planes and preserving sagittal Combined Flexion (CF).

**Methods:**

This retrospective study consecutively enrolled 360 patients who underwent primary TKA between October 2023 and October 2025 (199 in the manual group and 161 in the robotic group). Pre- and postoperative coronal and sagittal parameters were measured on standing full-length lower extremity radiographs and standard lateral radiographs. The “Tibial Cutting Guide Varus/Valgus Angle” (TCVA) was defined to quantify the deviation in cutting guide placement. The study adopted a systematic stepwise analysis strategy: initially detecting potential inter-planar coupling via correlation analysis, subsequently identifying independent predictors of sagittal posterior tibial slope using linear regression models, and finally calculating the equivalent rotational mismatch angle driving this coupling from regression coefficients based on stereometric projection principles.

**Results:**

In the M-TKA group, ΔMPTA showed a significant negative correlation with ΔPTS (*r* = − 0.209, *p* = 0.003), an effect not observed in the R-TKA group. TCVA was a significant independent predictor of postoperative PTS in M-TKA, and geometric analysis revealed an equivalent rotational mismatch of approximately 12.4° between the cutting guide rotational axis and the prosthesis placement axis. The CF preservation rate was significantly higher in the R-TKA group than in the M-TKA group (60.9% vs. 22.6%, *p* < 0.001). Additionally, ΔDFF and ΔPTS showed a significant negative correlation within the R-TKA group, demonstrating a unique intra-sagittal compensatory mechanism.

**Conclusion:**

Conventional M-TKA exhibits an iatrogenic coronal-sagittal coupling effect, whereby coronal plane correction errors are projected onto the sagittal plane through inherent rotational mismatch and cutting guide tilt, with error magnitude positively correlated with deformity severity. R-TKA effectively decouples these two planes and better preserves the native sagittal geometric alignment through femoral-tibial synergistic adjustment.

**Level of evidence:**

Level III, a retrospective cohort study.

**Supplementary Information:**

The online version contains supplementary material available at 10.1186/s42836-026-00398-3.

## Introduction

Achieving precise reconstruction of both the coronal and sagittal planes within three-dimensional space is a pivotal objective in modern total knee arthroplasty (TKA). While the Coronal Plane Alignment of the Knee (CPAK) classification [1] and surgical robotics [2, 3] have catalyzed personalized coronal alignment [4, 5], the sagittal plane remains critical yet underexplored. In particular, the recently proposed concept of “Combined Flexion” (CF) [6, 7] has provided a novel metric for the quantitative assessment of sagittal plane flexion–extension function. Still, its behavior during surgical reconstruction has not been fully investigated. Previous studies suggest that the coronal and sagittal parameters of the native knee are anatomically independent [8–11]. However, it remains unclear whether this “anatomical independence” is preserved during surgical reconstruction.

Conventional manual TKA (M-TKA) predominantly relies on extramedullary alignment for tibial positioning, which is inherently challenged by tibial torsion and ankle anatomical variations [12–14]. These variations can create a substantial geometric discrepancy between the rotational reference axis of the extramedullary guide rod and the final prosthesis placement axis. Furthermore, anatomical landmark variations [15, 16] and the inherent limitations of extramedullary alignment systems [17] often cause unintended coronal plane tilt of the cutting guide. We hypothesize that when this cutting guide tilt coexists with the inherent rotational discrepancy, it disrupts planar independence, triggering a latent “iatrogenic coronal-sagittal coupling effect.” This effect projects coronal plane operative errors onto the sagittal plane, causing the posterior tibial slope (PTS) to deviate unexpectedly and resulting in a loss of CF.

Currently, research on sagittal CF remains relatively limited and is primarily focused on robotic-assisted TKA (R-TKA), which offers three-dimensional planning and real-time feedback capabilities [6, 7]. The specific magnitude and mechanism of this hypothesized coupling effect in M-TKA remain a research blind spot. Moreover, without data from an M-TKA baseline, the true benefit of robotic technology in breaking this coupling remains unquantified. Therefore, this retrospective study aims to quantify the “coronal-sagittal coupling effect” in manual operations, explore its underlying mechanism, and evaluate the potential benefits of robotic technology in maintaining planar independence and preserving CF.

## Methods

### Patient selection

This retrospective study was approved by the Ethics Committee of China-Japan Friendship Hospital (Approval No.: 2023-KY-089), and informed consent was obtained from all patients. A total of 408 consecutive patients who underwent primary TKA at our institution between October 2023 and October 2025 were initially enrolled.

Inclusion criteria: (1) Diagnosis of primary knee osteoarthritis (Kellgren–Lawrence grade III-IV); (2) Significant varus or valgus deformity of the knee; (3) Availability of complete preoperative and postoperative standing full-length lower extremity radiographs and standard lateral radiographs.

**Fig. 1:**
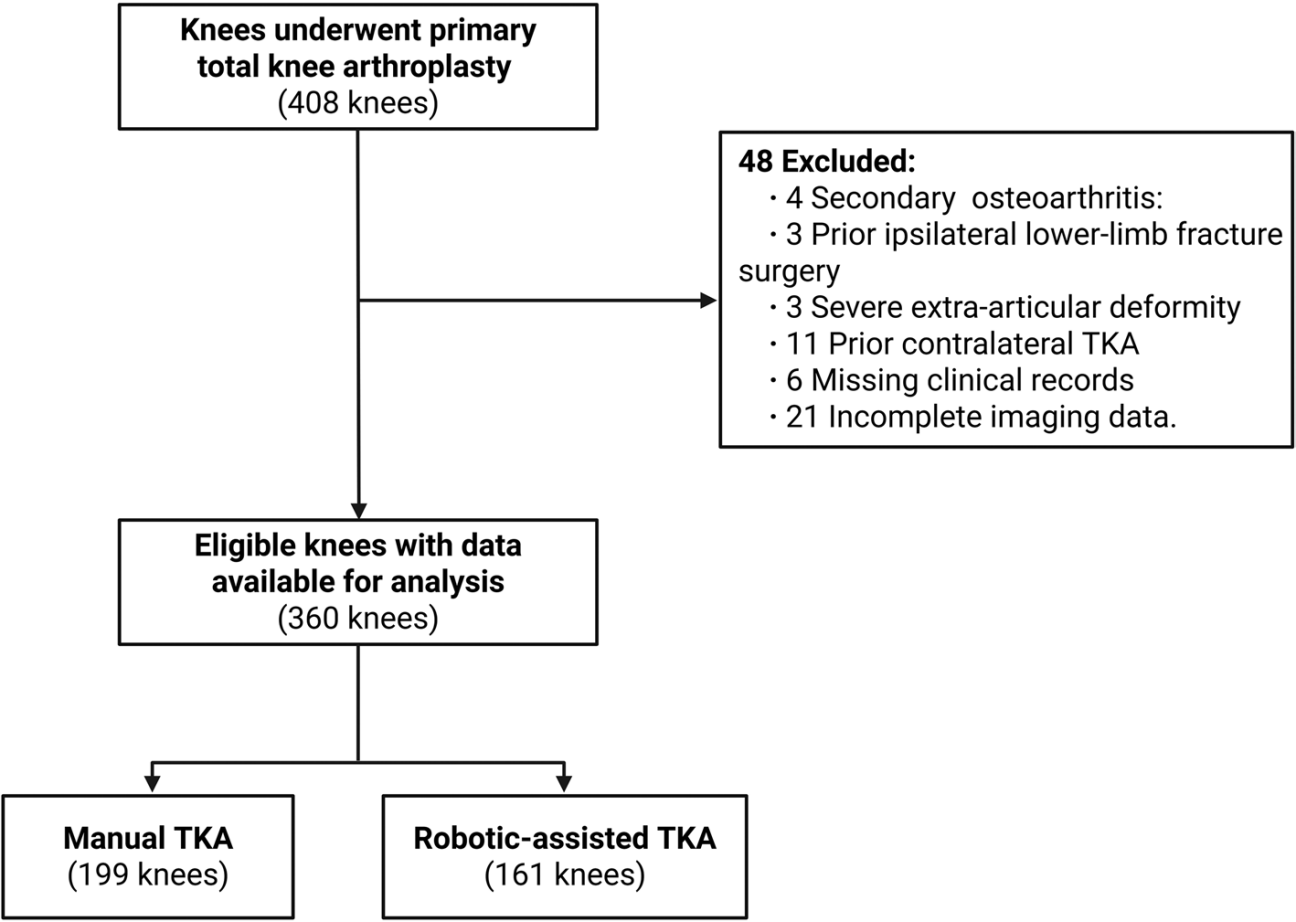
Flow diagram of patient enrollment

Exclusion criteria: (1) Secondary knee osteoarthritis; (2) History of previous ipsilateral lower extremity fracture surgery; (3) Severe extra-articular deformity; (4) History of previous contralateral knee arthroplasty; (5) Incomplete medical records or poor-quality radiographic images (e.g., inadequate overlap of the femoral condyles on lateral views, insufficient tibial length visualization). Ultimately, 360 cases were included in the study (Fig. 1).

### Surgical technique

Ll surgeries were performed by the same surgical team with extensive experience in both robotic-assisted and manual TKA. Preoperatively, patient allocation to either the R-TKA or M-TKA group was primarily determined by patient preference following comprehensive counseling. However, specific anatomical criteria mandated the use of conventional M-TKA. Patients presenting with extremely severe or complex deformities were exclusively assigned to the M-TKA group, including: (1) severe fixed flexion contractures (> 20°) that remained uncorrectable under anesthesia; (2) severe varus or valgus deformities combined with significant medial or lateral tibial plateau bone defects necessitating the intraoperative use of augments or stem extensions; and (3) severe deformities combined with medial or lateral collateral ligament insufficiency requiring the use of constrained prostheses. Intraoperatively, the decision to use a Cruciate-Retaining (CR) or Posterior-Stabilized (PS) prosthesis (Triathlon, Stryker) was made based on the integrity of the Posterior Cruciate Ligament (PCL) and the flexion gap balance.

The R-TKA group utilized a CT-based robotic navigation system (Mako, Stryker). The standard registration and execution workflow followed our previously published protocols [18, 19]. A Functional Alignment (FA) strategy was employed intraoperatively, with prosthesis positioning dynamically adjusted based on real-time soft tissue tension curves. For coronal and rotational parameters, adjustments were made to maintain gap balance while preserving the native joint line as much as possible. For sagittal parameters, femoral component flexion was allowed up to a maximum of 7° to accommodate the femoral anterior bow and optimize prosthesis fit, while avoiding extension. The target PTS was set at 5° for CR prostheses and 3° for PS prostheses, with fine-tuning permitted within a safe range to balance the flexion gap. To prevent wear or instability caused by extreme component positioning, strict safety boundaries were established during preoperative planning (Table 1).

**Table 1:** Predefined safety boundaries for component positioning in robotic-assisted total knee arthroplasty

Plane	Parameter	Reference System	Target/Safety Boundary
Coronal	Hip-Knee-Ankle Angle	MA	0° ± 3° (Varus/Valgus)
	Femoral Component	MA	0° ± 2° (Varus/Valgus)
	Tibial Component	MA	0° ± 2° (Varus/Valgus)
Sagittal	Distal Femoral Flexion	Distal Femoral MA	0° − 7° (Target: fit anatomy, usually 2° − 4°; No Extension)
	Posterior Tibial Slope	Proximal Tibial MA	CR: Target 5° (Range 3° − 7°) PS: Target 3° (Range 0° − 3°)
Rotational	Femoral Component	TEA	Dynamic adjustment to avoid anterior notching & balance gaps
	Tibial Component	Tibial AP Axis	0° ± 5° (Aligned to CT-planned axis)

Values represent target angles and acceptable ranges for each parameter

Positive values for the coronal plane indicate varus; negative values indicate valgus. For the sagittal plane, positive values indicate flexion or posterior slope

*MA* mechanical axis, *CR* cruciate-retaining, *PS* posterior-stabilized, *TEA* transepicondylar axis, *AP* anteroposterior

The M-TKA group underwent osteotomy using standard manual instrumentation, following the principles of Mechanical Alignment (MA). On the femoral side, the medullary canal was entered 1 cm anterior to the PCL insertion and beneath the anterior femoral cortex. An intramedullary alignment guide (set at 6° valgus) was inserted to perform the distal femoral resection. Femoral rotational alignment was referenced to the Transepicondylar Axis (TEA), with the 4-in-1 cutting block positioned at 3° external rotation to complete the anterior and posterior condylar resections. On the tibial side, an extramedullary alignment system was used. The proximal end of the alignment rod was aimed at the medial one-third of the tibial tuberosity, and the distal end was directed toward a point 3–5 mm medial to the ankle center. The tibial cutting guide was then installed to perform the proximal tibial resection perpendicular to the tibial mechanical axis in the coronal plane and with a 3° (PS) or 5° (CR) posterior slope in the sagittal plane. For both R-TKA and M-TKA, the rotational alignment of the tibial component was referenced to the Akagi line (the line connecting the midpoint of the patellar tendon to the center of the PCL tibial insertion).

### Radiographic assessment and parameter definitions

All radiographic measurements were independently performed by two orthopedic surgeons who were blinded to patient group allocation. To assess measurement reproducibility, each observer performed two independent measurements on the same radiograph at a 2-week interval, and the mean of these measurements was taken as the observer’s final measurement. When the interobserver measurement discrepancy exceeded 3°, a third senior surgeon joined the discussion to reach consensus. Intra-observer and inter-observer reliability were evaluated using the Intraclass Correlation Coefficient (ICC). Coronal plane parameters were measured using Matlab software (MathWorks, Natick, MA, USA); sagittal plane parameters were measured using a combination of RadiAnt DICOM Viewer (Medixant, Poland), ImageJ (National Institutes of Health, USA), and Matlab software (Supplementary Material 1).

#### Coronal parameters

**Fig. 2:**
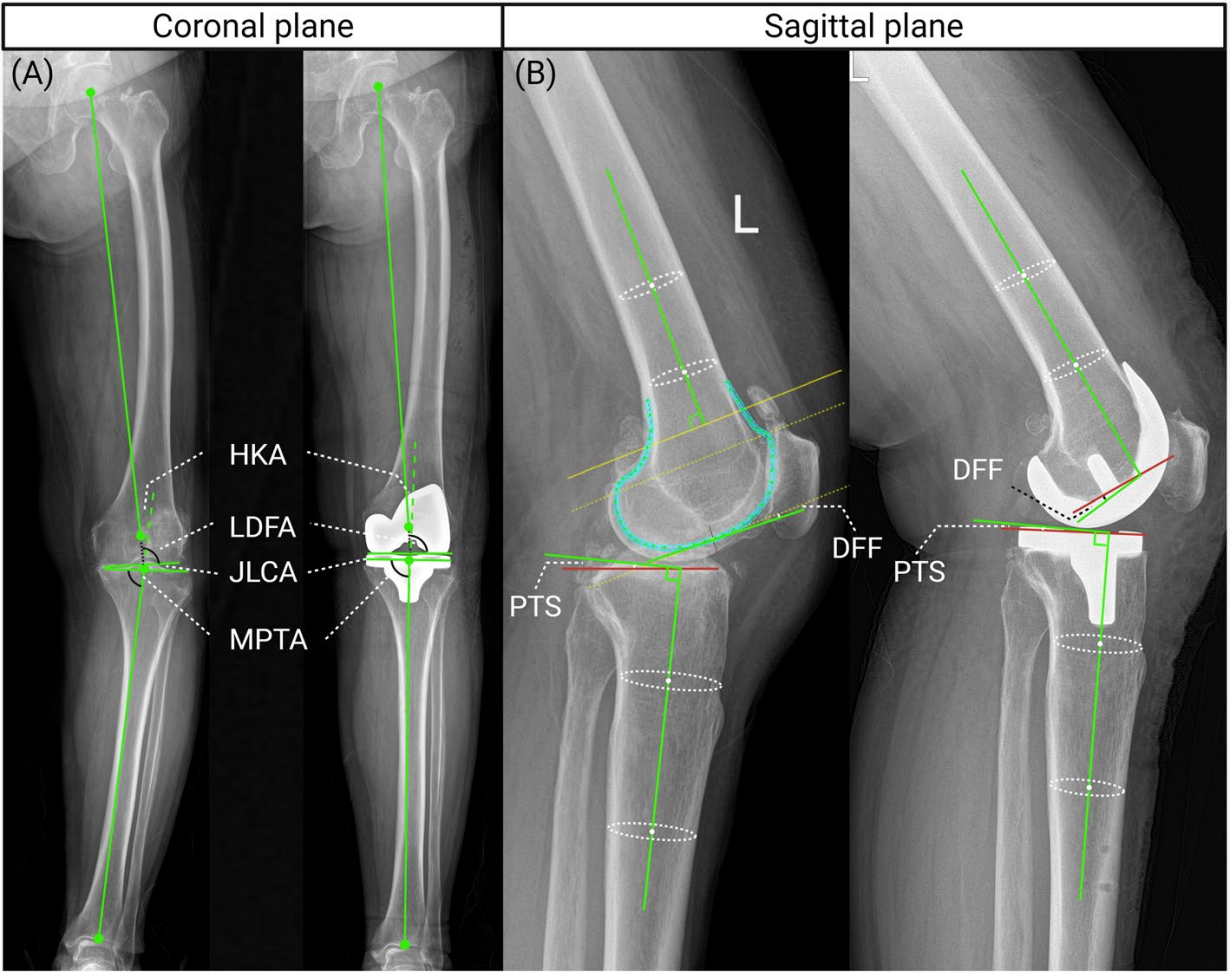
Radiographic measurement of coronal and sagittal plane parameters. **A** Coronal plane parameters measured on standing full-length lower extremity radiographs, including hip-knee-ankle angle (HKA), lateral distal femoral angle (LDFA), medial proximal tibial angle (MPTA), and joint line congruency angle (JLCA). **B** Sagittal plane parameters measured on standard lateral knee radiographs with adequate femoral condyle overlap, including posterior tibial slope (PTS) and distal femoral flexion (DFF)

Coronal parameters were measured on preoperative and postoperative standing full-length lower extremity radiographs (Fig. 2). The measured parameters included: Hip-Knee-Ankle angle (HKA), Lateral Distal Femoral Angle (LDFA), Medial Proximal Tibial Angle (MPTA), Joint Line Obliquity (JLO = MPTA + LDFA), and arithmetic Hip-Knee-Ankle angle (aHKA = MPTA − LDFA). The specific measurement methods are described in our previously published literature [18]. Additionally, this study defined the Tibial Cutting Guide Varus/Valgus Angle (TCVA) = 90° − postoperative MPTA, to quantify the coronal plane deviation of the tibial cutting guide relative to the mechanical axis in manual TKA. A positive value indicates varus, and a negative value indicates valgus.

#### Sagittal parameters

Sagittal parameters were measured on standard lateral radiographs, which required adequate overlap of the posterior borders of the medial and lateral femoral condyles.

##### Posterior tibial slope (PTS)

The angle between the tangent line of the proximal tibial articular surface and the perpendicular to the proximal tibial anatomical axis. A positive value indicates posterior slope, and a negative value indicates anterior slope.

##### Distal femoral flexion (DFF)

Preoperatively, this was defined as the angle between the femoral anatomical axis and the perpendicular to the tangent line of the distal articular surface; postoperatively, it was defined as the angle between the perpendicular to the femoral anatomical axis and the distal plane of the prosthesis. A positive value indicates flexion, and a negative value indicates extension.

##### Combined flexion (CF)

Defined as the sum of DFF and PTS (CF = DFF + PTS).

##### Coronal phenotype preservation

JLO and aHKA were calculated from preoperative and postoperative parameters according to the CPAK classification system. If the postoperative classification remained consistent with the preoperative classification, it was defined as “Preserved”; if the classification changed, it was defined as “Changed.”

##### Sagittal CF preservation

**Fig. 3:**
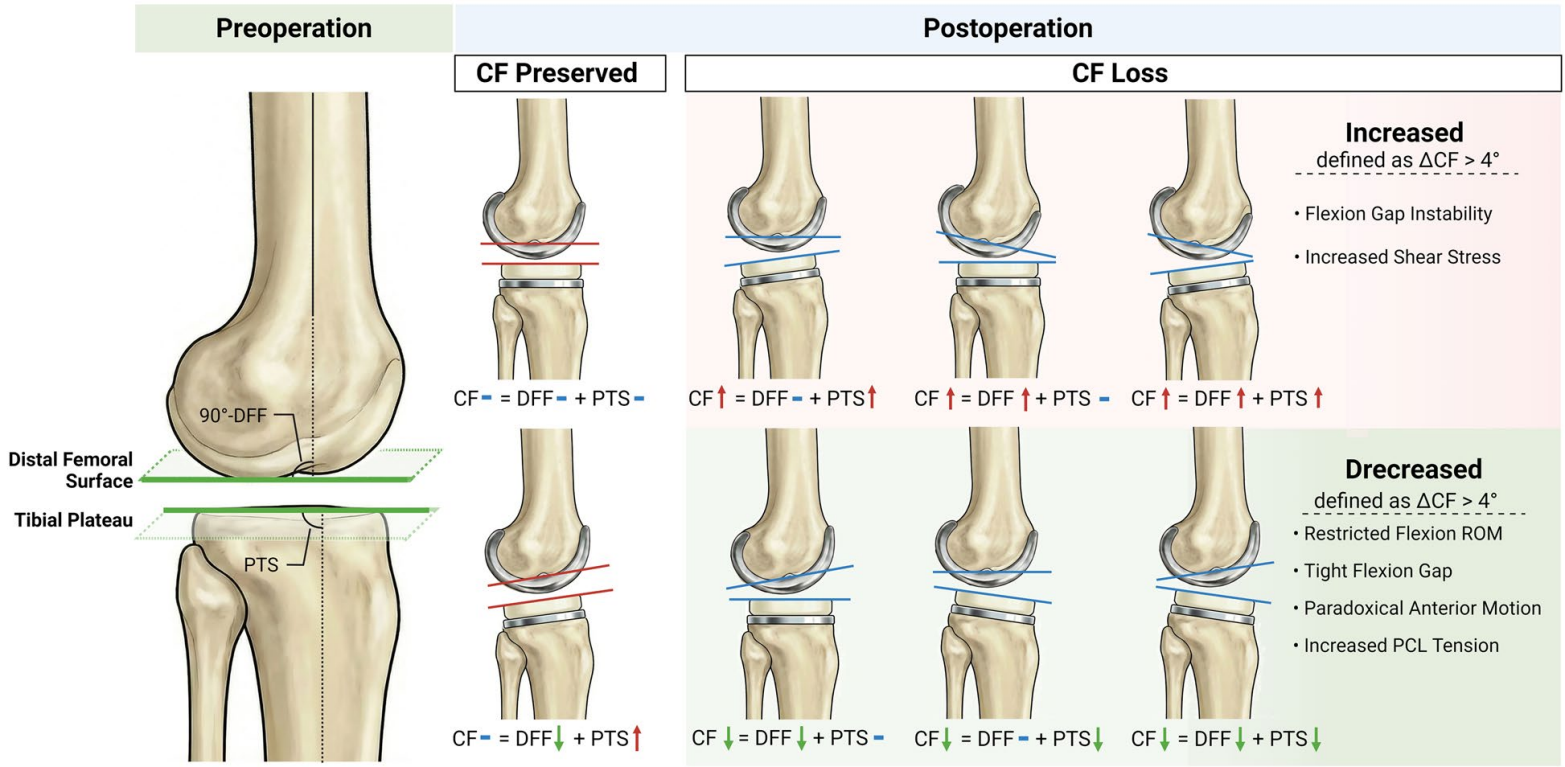
Schematic illustration of combined flexion (CF) preservation and loss. Blue horizontal lines indicate unchanged values, green arrows denote a decrease, and red arrows signify an increase. CF preservation was defined as an absolute postoperative change of 4° or less (|ΔCF| ≤ 4°), while CF loss was defined as a change exceeding this threshold (|ΔCF| > 4°). The left panel demonstrates a case of CF preservation, where the postoperative CF remains within the acceptable range of the preoperative value. The right panel illustrates CF loss, in which the postoperative CF exceeds the 4° threshold

This was defined as an absolute difference between postoperative CF and preoperative CF of no more than 4° (|ΔCF| ≤ 4°), a threshold informed by machine-learning–derived sagittal alignment targets associated with achieving minimal clinically important differences [7, 20]. Cases meeting this criterion were defined as “CF Preserved”; otherwise, they were defined as “CF Loss.” (Fig. 3).

### Statistical analysis

Data analysis was performed using SPSS 26.0 software (IBM Corp, Armonk, NY, USA). Continuous variables were expressed as mean ± standard deviation, and inter-group comparisons were conducted using an independent samples t-test. Categorical variables were expressed as frequencies and compared using the chi-square test. Pearson correlation analysis was used to evaluate the linear relationships between variables. Multiple linear regression models were constructed to identify independent predictors of sagittal plane parameter changes. Measurement reliability was assessed using the ICC (ICC > 0.8 was considered excellent agreement). A two-tailed *p* < 0.05 was considered statistically significant.

## Results

### Baseline characteristics and preoperative anatomy

The intra- and inter-observer ICC values for coronal plane parameters (HKA, MPTA, LDFA, JLCA) and PTS were > 0.85. For DFF measurement on lateral radiographs, the ICC values ranged from 0.82 to 0.94 (Supplementary Tables S1 and S2). A total of 360 patients were included (Fig. 1). There were no significant differences in demographic characteristics or preoperative coronal and sagittal parameters between the R-TKA (*n* = 161) and M-TKA (*n* = 199) groups (Table 2).

**Table 2:** Baseline demographic and radiographic characteristics of patients in the robotic-assisted and manual TKA groups

	**R-TKA (*n* = 161)**	**M-TKA (*n* = 199)**	***p-*value**
Age (years)	68.63 ± 6.45	67.82 ± 6.93	0.26
Laterality (left/right)	81/80	107/92	0.51
Implant type (CR/PS)	114/47	129/70	0.28
Gender (male/female)	30/131	45/154	0.36
BMI (kg/m^2^)	27.24 ± 3.77	27.31 ± 4.07	0.92
height (cm)	161.48 ± 6.85	160.47 ± 7.02	0.28
weight (kg)	71.19 ± 11.71	70.48 ± 12.35	0.66
**Coronal parameters**			
HKA (°)	9.12 ± 6.11	9.47 ± 6.48	0.60
LDFA (°)	89.25 ± 2.93	89.27 ± 3.00	0.97
MPTA (°)	85.06 ± 2.91	84.55 ± 3.04	0.11
JLCA (°)	4.95 ± 2.88	5.08 ± 2.88	0.66
aHKA (°)	− 4.19 ± 4.64	− 4.72 ± 4.81	0.29
JLO (°)	174.31 ± 3.53	173.81 ± 3.66	0.19
**Sagittal parameters**			
DFF (°)	0.25 ± 3.69	0.67 ± 3.64	0.28
PTS (°)	10.47 ± 3.40	10.78 ± 3.92	0.16
CF (°)	10.76 ± 4.90	11.46 ± 5.36	0.10

### Postoperative changes in coronal and sagittal alignment parameters

Post-hoc power analysis based on ΔPTS confirmed sufficient statistical power (> 99%). Postoperatively, coronal alignment parameters in both groups improved significantly and trended toward neutral (Table 3). Notably, the mean TCVA in the M-TKA group was 0.34° ± 2.16°, objectively quantifying the execution deviation unavoidable with conventional extramedullary alignment systems. In contrast, R-TKA employs an individualized osteotomy in which preoperative planning and robotic-arm assistance together eliminate TCVA at its origin. Furthermore, the R-TKA group demonstrated significantly higher preservation rates for both overall lower limb alignment (aHKA: 69.6% vs. 58.8%, *p* = 0.035) and joint line obliquity (JLO: 23.6% vs. 14.1%, *p* = 0.020).

**Table 3:** Postoperative coronal and sagittal plane parameters and their changes from baseline

	**R-TKA (*n* = 161)**	**M-TKA (*n* = 199)**	***p***-**value**
**Coronal parameters**			
HKA (°)	0.75 ± 2.92	2.64 ± 3.68	< 0.01
Δ (°)	− 8.37 ± 5.27	− 6.82 ± 5.80	< 0.01
LDFA (°)	90.91 ± 1.93	92.34 ± 2.55	< 0.01
Δ (°)	1.66 ± 2.76	3.07 ± 2.57	< 0.01
MPTA (°)	90.10 ± 1.74	89.66 ± 2.16	0.04
Δ (°)	5.04 ± 2.88	5.12 ± 3.19	N.s.
JLCA (°)	0.27 ± 0.86	0.31 ± 1.07	N.s.
Δ (°)	− 4.68 ± 2.94	− 4.77 ± 3.08	N.s.
aHKA (°)	− 0.81 ± 2.59	− 2.68 ± 3.32	< 0.01
Δ (°)	3.38 ± 4.07	2.04 ± 3.98	< 0.01
JLO (°)	181.01 ± 2.61	182.00 ± 3.36	< 0.01
Δ (°)	6.70 ± 3.91	8.19 ± 4.21	< 0.01
**Sagittal parameters**			
DFF (°)	2.59 ± 3.33	0.51 ± 3.67	< 0.001
Δ (°)	2.34 ± 4.77	− 0.17 ± 4.81	< 0.001
PTS (°)	3.30 ± 1.96	5.12 ± 2.65	< 0.001
Δ (°)	− 6.78 ± 3.87	− 5.66 ± 4.41	0.01
CF (°)	5.90 ± 3.88	5.62 ± 4.72	N.s.
Δ (°)	− 4.47 ± 5.75	− 5.83 ± 6.71	0.03

Values are presented as mean ± standard deviation.“Δ” indicates the change from preoperative to postoperative values (Δ = postoperative − preoperative). N.s., not significant. *p*-values were calculated using an independent samples t-test

**Fig. 4:**
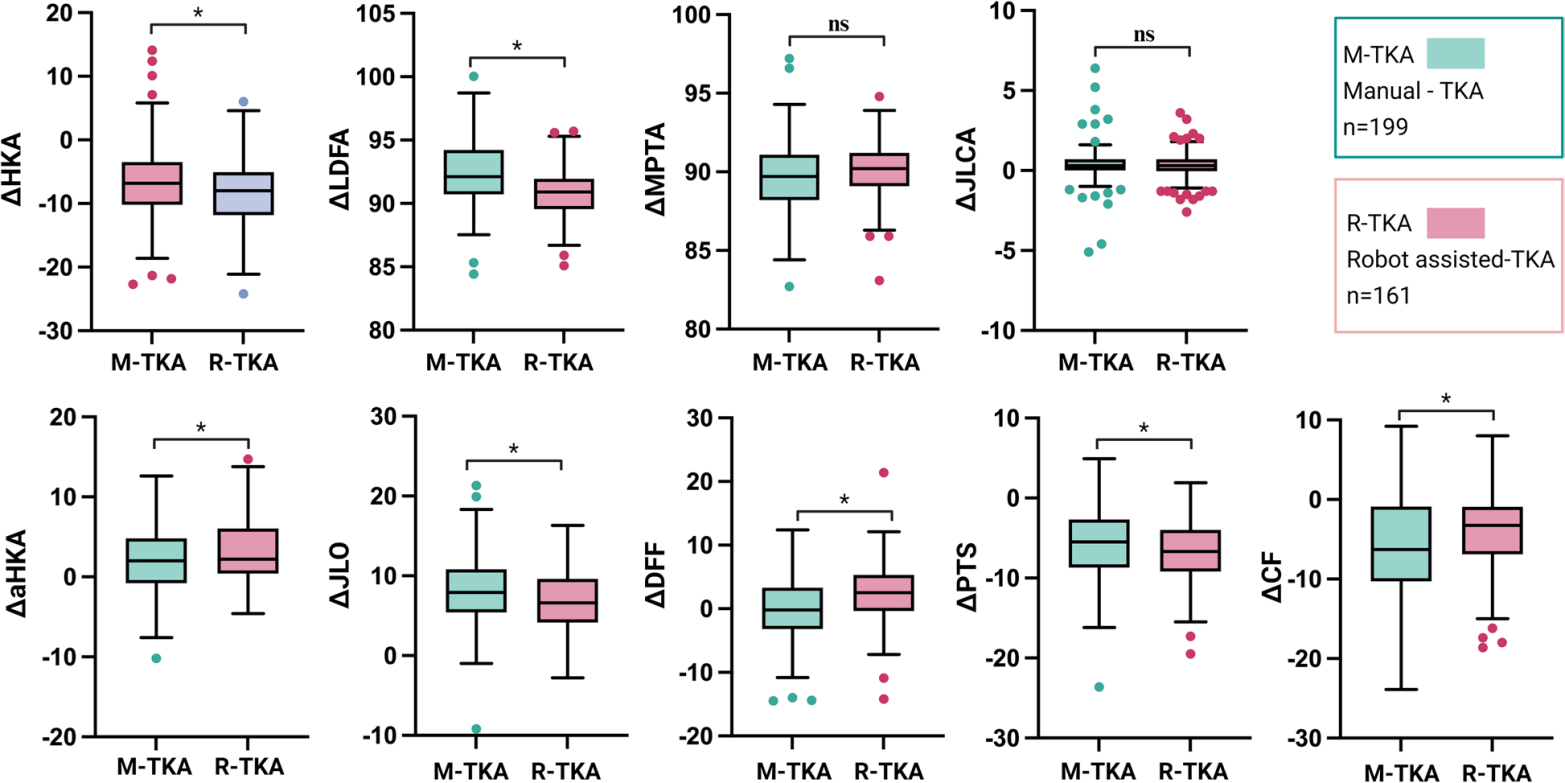
Comparison of changes in coronal and sagittal plane parameters between robotic-assisted and manual TKA groups. Error bars represent standard deviation. Significant differences between groups are indicated by asterisks (*p < 0.05)

Postoperatively, mean PTS decreased significantly in both groups, with the R-TKA group actually experiencing a greater reduction (ΔPTS: − 6.78° vs. − 5.66°, *p* = 0.01). Notably, this greater PTS reduction in the R-TKA group was accompanied by a significant increase in DFF (ΔDFF: 2.34° vs. − 0.17°, *p* < 0.001), resulting in a significantly smaller overall change in CF (ΔCF: − 4.47° vs. − 5.83°, *p* = 0.03) and a substantially higher CF preservation rate (60.9% vs. 22.6%, *p* < 0.01) (Fig. 4). In the sagittal plane, no significant baseline differences existed between the two groups.

### Impact of coronal strategy on sagittal preservation: from phenotype decomposition to geometric quantification

**Fig. 5:**
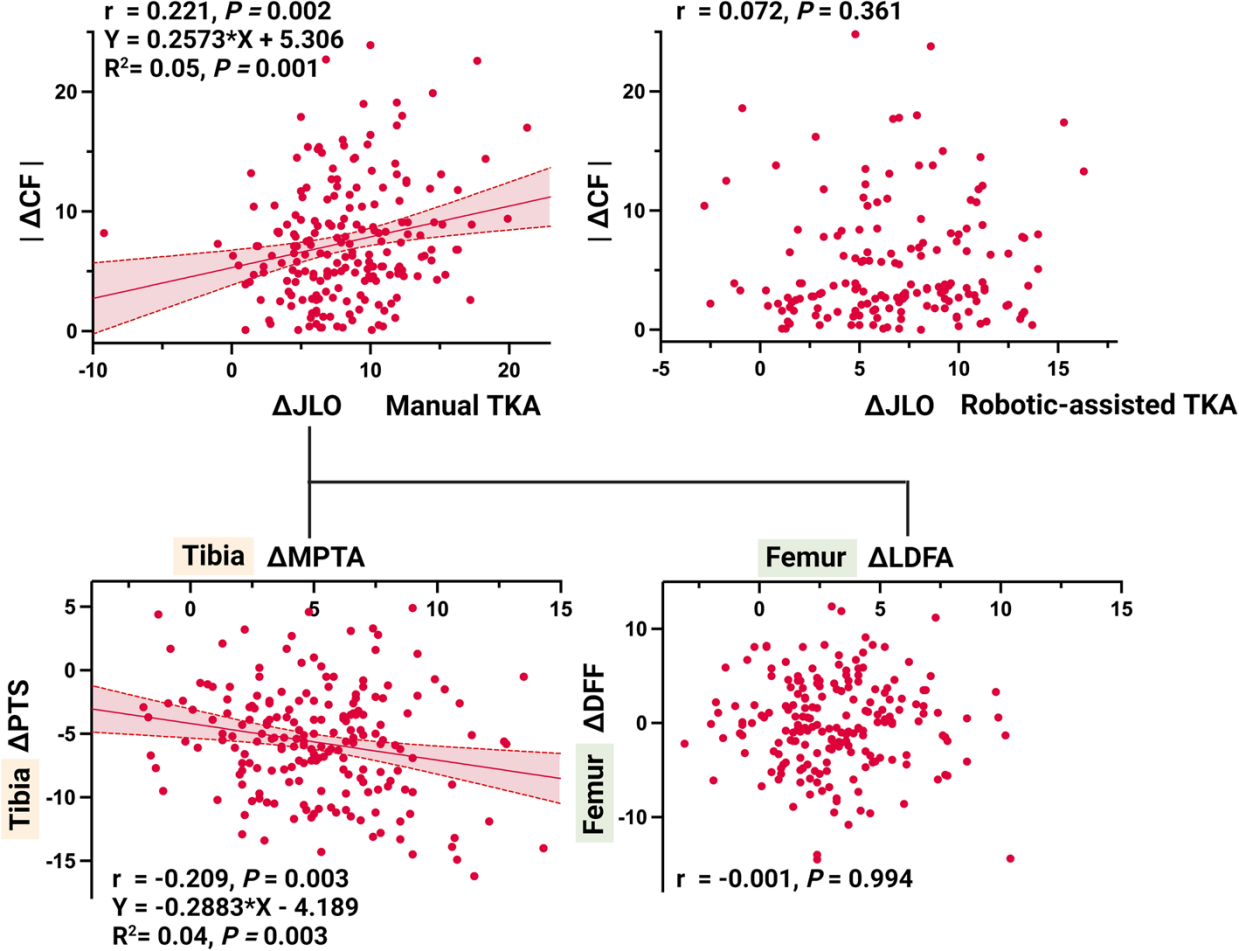
Correlation analysis of coronal-sagittal coupling: from composite parameters to bone-specific decomposition

In the M-TKA group, a significant coronal-sagittal coupling pattern was identified. At the composite-parameter level, ΔJLO was negatively correlated with ΔCF (*r* = − 0.238, *p* = 0.003), whereas ΔaHKA showed no significant association with ΔCF (*p* > 0.05). Decomposition into bone-specific parameters (JLO = MPTA + LDFA; CF = PTS + DFF) localized this coupling to the tibial side: ΔMPTA was significantly negatively correlated with ΔPTS (*r* = − 0.209, *p* = 0.003, Table 4); this association remained significant after adjustment for age, sex, laterality, preoperative PTS, and preoperative MPTA (partial *r* = − 0.173, *p* = 0.015; Supplementary Table S3), while no femoral coronal-sagittal correlations were observed (Fig. 5).

**Table 4:** Correlation analysis between changes in coronal and sagittal plane parameters in robotic-assisted and manual TKA groups

			**ΔHKA**	**ΔMPTA**	**ΔLDFA**	**ΔJLCA**	**ΔaHKA**	**ΔJLO**
**M-TKA**	ΔDFF	*r*	0.121	− 0.097	− 0.001	0.087	− 0.078	− 0.074
		*p*	0.090	0.171	0.994	0.220	0.275	0.298
	ΔPTS	*r*	− 0.003	**− 0.209**	− 0.128	− 0.050	− 0.036	**− 0.282**
		*p*	0.963	**0.003**	0.072	0.486	0.610	**< 0.001**
	ΔCF	*r*	0.084	**− 0.207**	− 0.134	0.030	− 0.080	**− 0.238**
		*p*	0.237	**0.003**	0.060	0.675	0.264	**0.001**
**R-TKA**	ΔDFF	*r*	0.007	0.096	0.065	0.041	0.024	0.117
		*p*	0.929	0.226	0.410	0.606	0.767	0.140
	ΔPTS	*r*	0.016	− 0.150	− 0.057	− 0.039	− 0.068	− 0.151
		*p*	0.844	0.057	0.476	0.627	0.392	0.057
	ΔCF	*r*	0.012	− 0.020	0.018	0.001	− 0.026	− 0.002
		*p*	0.878	0.803	0.821	0.988	0.742	0.981

Values represent Pearson correlation coefficients (*r*) and corresponding *p*-values. Statistically significant results are shown in bold

To investigate the mechanism underlying this tibial-side coupling, we analyzed the relationship between TCVA and postoperative PTS in the M-TKA group. TCVA was positively correlated with postoperative PTS (*r* = 0.178, *p* = 0.012) and remained a significant independent predictor in multiple linear regression (B = 0.219, *p* = 0.012). According to the principle of geometric projection, the regression coefficient B represents the tangent of the angular mismatch through which coronal plane error is projected onto the sagittal plane. Geometric transformation of this coefficient, therefore, yields:

$$\uptheta=\arctan\left(\left|\mathrm B\right|\right)=\arctan\left(0.219\right)\approx12.4^\circ$$

**Fig. 6:**
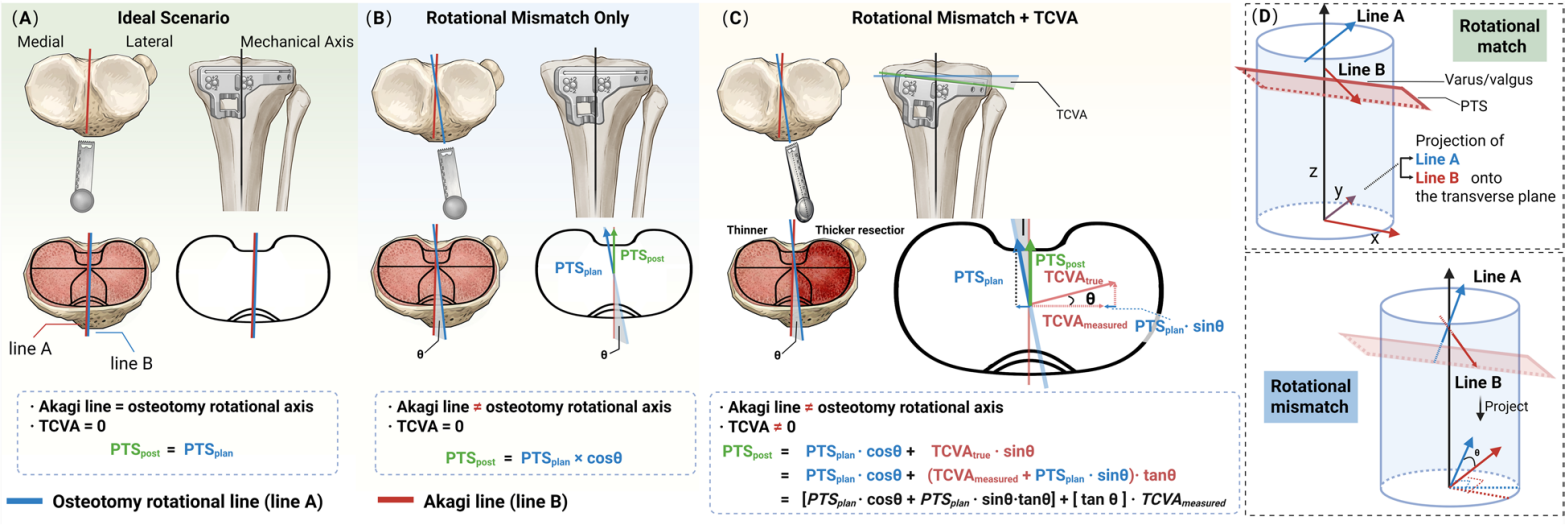
Schematic illustration of the geometric mechanism underlying iatrogenic coronal-sagittal coupling in tibial osteotomy. **A** Ideal scenario: the osteotomy rotational axis coincides with the Akagi line and the cutting guide is perpendicular to the mechanical axis (TCVA = 0°). **B** Rotational mismatch only: the osteotomy rotational axis diverges from the Akagi line by angle θ, but the cutting guide remains correctly aligned. **C** Rotational mismatch combined with cutting guide tilt: coronal deviation (TCVA ≠ 0°) is projected onto the sagittal plane through the rotational mismatch, causing unintended PTS alteration. **D** The geometric projection model is used to calculate the equivalent rotational mismatch angle from regression coefficients. Abbreviations: PTS_post, postoperative posterior tibial slope; PTS_plan, planned posterior tibial slope; TCVA, tibial cutting guide varus/valgus angle. A detailed derivation of the geometric projection equations and the estimation of θ is provided in Supplementary Material 2

This indicates an equivalent rotational mismatch of approximately 12.4° of external rotation between the osteotomy reference axis of the extramedullary guide rod (medial one-third of the tibial tuberosity to the point medial to the ankle center) and the prosthesis placement reference axis (Akagi line) (Fig. 6). This estimate was essentially unchanged after further adjustment for age, sex, laterality, preoperative PTS and preoperative MPTA (adjusted B = 0.224, *p* = 0.016; arctan(0.224) ≈ 12.6°; Supplementary Table S4), supporting a direct geometric mechanism rather than confounding.

In contrast, the R-TKA group showed no significant correlations between any coronal and sagittal parameter changes, whether at the composite or bone-specific level (Table 4), and multiple linear regression confirmed that none of the coronal variables significantly predicted ΔPTS (all *p* > 0.05), confirming effective coronal-sagittal decoupling. Notably, within the sagittal plane, ΔDFF was significantly negatively correlated with ΔPTS (*r* = − 0.356, *p* < 0.05), revealing an intra-sagittal compensatory mechanism unique to the robotic group that was absent in M-TKA.

## Discussion

The principal finding of this study is that the inherent 12.4° rotational mismatch and cutting guide tilt in M-TKA may induce an “iatrogenic coronal-sagittal coupling effect”, whereby coronal osteotomy errors are projected onto the sagittal plane. This implies that aggressive correction of severe coronal deformities carries the risk of disrupting the sagittal geometric alignment, potentially leading to altered flexion gaps, increased joint shear stress, or restricted postoperative range of motion. These findings also remind us that the three-dimensional limitations of conventional extramedullary alignment systems must be fully recognized in clinical practice. When performing manual TKA in patients requiring substantial coronal correction, surgeons should remain highly vigilant and minimize cutting guide tilt. In contrast, R-TKA not only achieves precise decoupling of the two planes but also effectively preserves overall CF through a unique intra-sagittal synergistic compensatory mechanism. R-TKA may therefore be more actively incorporated into routine surgical practice, offering a more reliable option for achieving simultaneous and independent optimization of both coronal and sagittal alignment in complex deformity cases.

### A novel method for measuring preoperative DFF on lateral radiographs

Currently, very few studies have addressed preoperative DFF. We believe this is because preoperative DFF lacks a standardized and reliable measurement method, which has confined most sagittal plane research to PTS and hindered the development of personalized sagittal alignment studies. Therefore, we explored and validated a reliable method for measuring DFF on standard lateral radiographs using the distal femoral anatomical axis (ICC > 0.85). Although Chai et al. [21] reported that there is an average angle of approximately 2° between the distal femoral anatomical axis and the mechanical axis, which would result in DFF values being approximately 2° more extended compared to the traditionally recognized DFF, our study primarily focused on Δ values, thereby eliminating this interference. This method enabled us, for the first time, to quantitatively assess sagittal CF in a manual TKA cohort lacking preoperative CT data, thus providing a solid data foundation for comparative studies between robotic and manual techniques in three-dimensional reconstruction.

### Iatrogenic coronal-sagittal coupling in manual TKA

#### Geometric mechanism

Previous radiographic studies by Hiyama [8], Franceschetti [9], Corbett [22], and other scholars have confirmed that in the preoperative native knee, there is no correlation between coronal plane phenotype and sagittal PTS. To investigate whether intraoperative coronal plane adjustment is similarly independent of sagittal plane adjustment, we adopted a “whole-to-part” analytical strategy. Our results revealed a significant coronal-sagittal coupling phenomenon in the M-TKA group, and decomposition analysis further identified the interaction between tibial-side parameters as the core driver of the overall coupling (ΔJLO vs. ΔCF). Although the absolute value of the above correlation coefficient is relatively small (*r* ≈ − 0.2), this finding challenges the traditional notion that “the two planes do not interfere with each other under standard osteotomy procedures.” It also prompts us to consider more deeply: why do two theoretically orthogonal planes exhibit crosstalk in M-TKA clinical practice, while this coupling disappears in R-TKA?

We believe that this coupling does not originate from inherent mechanical design flaws in the osteotomy instrumentation itself. In fact, the cutting module of the standard extramedullary alignment system is designed according to the principle of orthogonality. When the cutting slot is locked, the coronal plane parameter and the sagittal plane parameter (PTS) should theoretically be established simultaneously and independently.

The fundamental causes of this coupling are twofold:

First, the inconsistency between the rotational axis of the tibial extramedullary guide rod and the prosthesis installation reference axis constitutes the geometric basis for this coupling (further details are provided in Supplementary Material 3). In M-TKA, the rotational positioning of the osteotomy depends on the placement of the extramedullary guide rod. Surgeons typically aim the proximal end at the medial one-third of the tibial tuberosity and its distal end at a point 3–5 mm medial to the ankle center, to establish the rotational axis [23, 24]. However, the final prosthesis installation is based on the Akagi line. Even if the surgeon performs the procedure flawlessly, these two lines do not completely coincide in three-dimensional space [25, 26]. This means that the “coronal plane” and “sagittal plane” parameters set during osteotomy will exhibit geometric projection errors relative to the actual parameters after prosthesis installation due to the difference in reference frames. To understand this principle more intuitively, we can illustrate with an extreme example: suppose the rotational axis of the extramedullary guide rod is completely offset, forming a 90° angle with the Akagi line (i.e., the guide rod is positioned directly on the medial side of the tibia). In this case, if the surgeon sets “3° varus, 7° posterior slope” for the osteotomy, then after the prosthesis is installed according to the Akagi line, the actual result will become 3° posterior slope and 7° varus—the coronal and sagittal plane parameters are completely interchanged. Of course, in actual surgery, the angle between the cutting guide rotational axis and the Akagi line would never reach such an extreme degree. Back-calculation from our data indicates that this “equivalent rotational mismatch angle” is approximately 12.4° of external rotation (Supplementary Material 4). If only the rotational mismatch exists and TCVA = 0, then ΔMPTA would not correlate with ΔPTS. In this scenario, the postoperatively measured PTS would relate to the surgeon-intended target set on the conventional cutting guide as: PTS_post = PTS_plan × cos(12.4°) ≈ PTS_plan × 0.98, representing only a 2% deviation from the planned value.

More importantly, the failure of the extramedullary guide rod to be placed parallel to the tibial mechanical axis is the direct cause of TCVA generation. Once TCVA is generated, it creates a component in the sagittal plane that is projected through the aforementioned rotational mismatch, thereby unexpectedly altering PTS. It is noteworthy that TCVA is not equivalent to ΔMPTA, which is intended to correct the deformity. However, our correlation analysis showed that TCVA was significantly correlated with both ΔMPTA and preoperative MPTA. This reveals that patients with more severe preoperative varus deformity are more likely to develop larger TCVA. The anatomical basis underlying this phenomenon is multifactorial. First, patients with more severe preoperative varus deformity often exhibit more pronounced anatomical variations, such as corresponding degrees of tibial tuberosity lateralization [27, 28] and medial soft tissue contracture. An important observational study indicated that when preoperative knee varus deformity exceeds 10°, structures such as the medial collateral ligament undergo intrinsic, progressive shortening [29]. Second, the extramedullary alignment system is designed based on the assumption that the tibia is an ideal linear structure; however, long-standing varus stress leads to bowing deformity of the tibial shaft [30]. This bowing deformity, together with the lateralized tibial tuberosity, causes the distal alignment point of the extramedullary guide rod to shift relatively medially while the proximal alignment point shifts relatively laterally, thereby geometrically inducing varus tilt of the proximal cutting guide. From an operative standpoint, patients with more severe preoperative varus deformity (who typically require greater ΔMPTA for correction) often have more severe bone defects and sclerotic bone on the medial plateau [31]. The hard sclerotic bone and irregular bone defect surfaces undoubtedly increase the difficulty of pin fixation for the extramedullary alignment guide, making the cutting guide more prone to coronal plane tilt (i.e., generating larger TCVA).

These factors form an interconnected cascading effect: greater preoperative deformity → more significant anatomical variations and bone changes → greater propensity for guide tilt (increased TCVA) → projection through the 12.4°rotational mismatch → ultimately leading to greater PTS loss.

#### Clinical consequences

PTS is a critical determinant of the flexion gap, knee joint stability, and posterior femoral rollback [32]. Extensive literature has documented the adverse biomechanical sequelae of PTS deviation: decreased PTS leads to reduced flexion gap, increased PCL tension, and diminished postoperative range of motion [33], while excessive PTS results in anterior tibial translation, flexion instability, and accelerated polyethylene wear [32, 34]. Among these, flexion instability represents one of the most prognostically unfavorable indications for revision surgery [35]. Importantly, our data demonstrate that patients with the most severe preoperative deformity, those requiring the greatest coronal correction, are paradoxically the most susceptible to large TCVA generation, placing them at the highest risk of sagittal geometric malalignment. This subgroup, therefore, warrants particular clinical vigilance.

#### Mitigation strategies

Based on the geometric mechanism elucidated in our study, we have outlined a tiered approach to mitigate the coupling effect within the manual surgical paradigm: (1) Minimizing TCVA generation through refined extramedullary guide positioning techniques, including centering the distal reference over the talus, compensatory valgus adjustment for thick lateral soft tissues, and reliance on proximal tibial landmarks when tibial bowing is present [30, 36]; (2) Intraoperative verification of cutting guide alignment prior to committing to the osteotomy, using fluoroscopy [37] or portable accelerometer-based navigation [38], given that up to 45% of manually positioned tibial guides have been shown to have unsatisfactory sagittal alignment [25]; (3) Computer-assisted navigation, which has been shown in meta-analyses to significantly reduce both coronal and sagittal alignment outliers [38, 39]. Although the above strategies can effectively reduce TCVA, they still cannot eliminate the second fundamental cause of the coupling, the inherent ~ 12.4° rotational mismatch between the extramedullary guide rod axis and the Akagi line.

### Reciprocal compensation of DFF and PTS in robotic-assisted TKA

In stark contrast to the M-TKA group, there was no significant correlation between any coronal plane parameter change and sagittal plane parameter change in the R-TKA group. This fully demonstrates that, based on a unified three-dimensional coordinate system derived from preoperative CT, R-TKA fundamentally eliminates the rotational reference frame mismatch. Furthermore, through the rigid constraints of optical navigation, it avoids the generation of execution errors, thereby completely severing this negative cascading effect (Supplementary Material 5).

The advantages of robotic technology extend far beyond passively “avoiding errors.” We found a significant negative correlation between ΔDFF and ΔPTS in the R-TKA group (*r* = − 0.356, *p* < 0.05), a relationship that did not exist in the M-TKA group. This reveals an important advantage under the robotic functional alignment philosophy: the system can not only independently control each parameter but also intelligently coordinate within the sagittal plane. When PTS is substantially reduced due to individualized needs (e.g., to match the flexion gap), the system can compensatorily increase femoral component flexion (DFF) to maintain overall sagittal CF. This reciprocal compensation mechanism is precisely the embodiment of the functional alignment philosophy’s pursuit of individualized functional reconstruction, restoring lower limb alignment while maximally preserving the patient’s inherent kinematic characteristics. Therefore, although the magnitude of PTS reduction in the R-TKA group was even slightly greater than that in the M-TKA group, its CF preservation rate (60.9%) was significantly superior owing to its precise femoral-side compensation, demonstrating superior preservation of the sagittal characteristics.

This study also has some limitations. First, although no significant differences were observed in the preoperative baseline characteristics between the two groups, selection bias cannot be entirely excluded given the retrospective, non-randomized design, and the findings therefore warrant further validation in prospective randomized controlled trials. Second, the study lacks long-term clinical functional follow-up and thus cannot directly relate differences in the CF preservation rate to actual patient function. Third, this was a single-center study, which may limit the generalizability of the findings to other institutions with different surgical volumes. Fourth, the R-TKA group was treated using a single image-based robotic platform (Mako, Stryker), and the findings may therefore not be directly generalizable to other robotic systems. Fifth, although the use of CR and PS implants was broadly balanced between groups, pooling two different implant designs within each group remains a potential source of confounding. Sixth, the superior sagittal compensation observed in the R-TKA group may be partially attributed to the Functional Alignment strategy, which inherently permits femoral flexion modifications, whereas conventional Mechanical Alignment restricts such adjustments; thus, the surgical tool and alignment philosophy remain confounded in this study. Finally, the coupling effect was quantified through regression analysis based on imaging data rather than direct intraoperative measurement; future validation using cadaveric experiments or navigation-based methods would further strengthen the robustness of these conclusions.

## Conclusion

This study is the first to reveal and quantify the “deformity-correlated coronal-sagittal coupling effect” present in conventional manual TKA, elucidating the core advantages of robotic technology in achieving three-dimensional precision alignment and preserving native sagittal characteristics. This provides a robust theoretical basis for surgical strategy selection when managing complex knee deformities.

## Supplementary Information

Supplementary Material 1 (Measurement Method for Distal Femoral Flexion). Supplementary Material 2 (Detailed Explanation of Figure 6). Supplementary Material 3 (Detailed mechanism of the mismatch between the osteotomy axis and the tibial component placement axis in manual TKA). Supplementary Material 4 (Analysis of the plausibility of a 12.4° angle between the osteotomy rotational axis and the Akagi line). Supplementary Material 5 (Demonstration of Robotic Decoupling) and Supplementary tables (Tables S1-S4).

## Data Availability

The datasets generated and/or analysed during the current study are available from the corresponding author on reasonable request.

## Abbreviations

aHKA: Arithmetic Hip-Knee-Ankle Angle
AP: Anteroposterior
BMI: Body Mass Index
CF: Combined Flexion
CI: Confidence Interval
CPAK: Coronal Plane Alignment of the Knee
CR: Cruciate-Retaining
CT: Computed Tomography
DFF: Distal Femoral Flexion
FA: Functional Alignment
HKA: Hip-Knee-Ankle Angle
ICC: Intraclass Correlation Coefficient
JLCA: Joint Line Congruency Angle
JLO: Joint Line Obliquity
LDFA: Lateral Distal Femoral Angle
MA: Mechanical Alignment
MPTA: Medial Proximal Tibial Angle
M-TKA: Manual Total Knee Arthroplasty
PCL: Posterior Cruciate Ligament
PS: Posterior-Stabilized
PTS: Posterior Tibial Slope
R-TKA: Robotic-Assisted Total Knee Arthroplasty
TCVA: Tibial Cutting Guide Varus/Valgus Angle
TEA: Transepicondylar Axis
TKA: Total Knee Arthroplasty
