# Supplementary material for: Iatrogenic coronal-sagittal coupling driven by a 12.4° rotational mismatch in manual total knee arthroplasty and precise decoupling with robotic assistance: a radiographic retrospective cohort study
Source: Arthroplasty. 2026 Jun 3;8:41. doi: 10.1186/s42836-026-00398-3 (PMC13231751; doi:10.1186/s42836-026-00398-3)
Supplement: Supplementary file 1 — Supplementary Material 1 (Measurement Method for Distal Femoral Flexion). Supplementary Material 2 (Detailed Explanation of Figure 6). Supplementary Material 3 (Detailed mechanism of the mismatch between the osteotomy axis and the tibial component placement axis in manual TKA). Supplementary Material 4 (Analysis of the plausibility of a 12.4° angle between the osteotomy rotational axis and the Akagi line). Supplementary Material 5 (Demonstration of Robotic Decoupling) and Supplementary tables (Tables S1-S4). [file 42836_2026_398_MOESM1_ESM.zip › supplementary material/supplementary table.docx]

**Supplementary Table S1**. Intra-observer ICC values for radiographic parameters.

|  | HKA | LDFA | MPTA | JLCA | DFF | PTS |
| --- | --- | --- | --- | --- | --- | --- |
| Preoperation | 0.955 | 0.917 | 0.952 | 0.891 | 0.862 | 0.894 |
| 95% CI | 0.930-0.988 | 0.899-0.935 | 0.939-0.962 | 0.859-0.917 | 0.831-0.889 | 0.872-0.913 |
| Postperation | 0.951 | 0.938 | 0.944 | 0.882 | 0.903 | 0.945 |
| 95% CI | 0.937-0.962 | 0.922-0.951 | 0.929-0.956 | 0.848-0.910 | 0.871-0.929 | 0.931-0.957 |

**Supplementary Table S2**. Inter-observer ICC values for radiographic parameters.

|  | HKA | LDFA | MPTA | JLCA | DFF | PTS |
| --- | --- | --- | --- | --- | --- | --- |
| Preoperation | 0.941 | 0.929 | 0.931 | 0.895 | 0.859 | 0.889 |
| 95% CI | 0.927-0.952 | 0.910-0.941 | 0.914-0.944 | 0.864-0.932 | 0.827-0.898 | 0.867-0.910 |
| Postperation | 0.929 | 0.905 | 0.912 | 0.885 | 0.899 | 0.941 |
| 95% CI | 0.915-0.940 | 0.884-0.920 | 0.894-0.925 | 0.835-0.905 | 0.863-0.935 | 0.928-0.953 |

**Supplementary Table S3.** Multiple linear regression analysis for ΔPTS in the M-TKA group (n = 199), adjusted for potential confounders.

*Model summary: R² = 0.6649, Adjusted R² = 0.6544, F = 63.49, P < 0.001*

| **Variable** | **B** | **SE** | **β** | **t** | **P** | **95% CI** | **VIF** |
| --- | --- | --- | --- | --- | --- | --- | --- |
| ΔMPTA | -0.224 | 0.092 | -0.162 | -2.429 | **0.0160** | [-0.405, -0.042] | 2.54 |
| Age | -0.015 | 0.027 | -0.023 | -0.556 | 0.5788 | [-0.068, 0.038] | 1.02 |
| Sex (male) | 0.568 | 0.458 | 0.054 | 1.239 | 0.2170 | [-0.336, 1.472] | 1.09 |
| Laterality (right) | 0.392 | 0.383 | 0.044 | 1.025 | 0.3067 | [-0.363, 1.148] | 1.08 |
| Preoperative PTS | -0.887 | 0.048 | -0.789 | -18.299 | **<0.001** | [-0.982, -0.791] | 1.06 |
| Preoperative MPTA | -0.255 | 0.096 | -0.176 | -2.646 | **0.0088** | [-0.445, -0.065] | 2.53 |

*Dependent variable: ΔPTS. B, unstandardized coefficient; SE, standard error; β, standardized coefficient; CI, confidence interval; VIF, variance inflation factor. Bold P values indicate statistical significance (P < 0.05). All VIF values < 5, indicating no multicollinearity.*

**Supplementary Table S4.** Multiple linear regression analysis for postoperative PTS with TCVA as the primary predictor in the M-TKA group (n = 199), adjusted for potential confounders.

*Model summary: R² = 0.0723, Adjusted R² = 0.0433, F = 2.49, P = 0.024*

| **Variable** | **B** | **SE** | **β** | **t** | **P** | **95% CI** |
| --- | --- | --- | --- | --- | --- | --- |
| TCVA | 0.224 | 0.092 | 0.182 | 2.429 | **0.0160** | [0.042, 0.405] |
| Age | -0.015 | 0.027 | -0.039 | -0.556 | 0.5788 | [-0.068, 0.038] |
| Sex (male) | 0.568 | 0.458 | 0.090 | 1.239 | 0.2170 | [-0.336, 1.472] |
| Laterality (right) | 0.392 | 0.383 | 0.074 | 1.025 | 0.3067 | [-0.363, 1.148] |
| Preoperative PTS | 0.113 | 0.048 | 0.167 | 2.336 | **0.0205** | [0.018, 0.209] |
| Preoperative MPTA | -0.032 | 0.067 | -0.036 | -0.473 | 0.6367 | [-0.163, 0.100] |

*Dependent variable: postoperative PTS. TCVA, Tibial Cutting Guide Varus/Valgus Angle. The adjusted regression coefficient of TCVA (B = 0.224) yields arctan(0.224) ≈ 12.6°, consistent with the unadjusted estimate of 12.4°. Bold P values indicate statistical significance (P < 0.05).*
