# Supplementary material for: Iatrogenic coronal-sagittal coupling driven by a 12.4° rotational mismatch in manual total knee arthroplasty and precise decoupling with robotic assistance: a radiographic retrospective cohort study
Source: Arthroplasty. 2026 Jun 3;8:41. doi: 10.1186/s42836-026-00398-3 (PMC13231751; doi:10.1186/s42836-026-00398-3)
Supplement: Supplementary file 1 — Supplementary Material 1 (Measurement Method for Distal Femoral Flexion). Supplementary Material 2 (Detailed Explanation of Figure 6). Supplementary Material 3 (Detailed mechanism of the mismatch between the osteotomy axis and the tibial component placement axis in manual TKA). Supplementary Material 4 (Analysis of the plausibility of a 12.4° angle between the osteotomy rotational axis and the Akagi line). Supplementary Material 5 (Demonstration of Robotic Decoupling) and Supplementary tables (Tables S1-S4). [file 42836_2026_398_MOESM1_ESM.zip › supplementary material/supplementary material 2.docx]

**
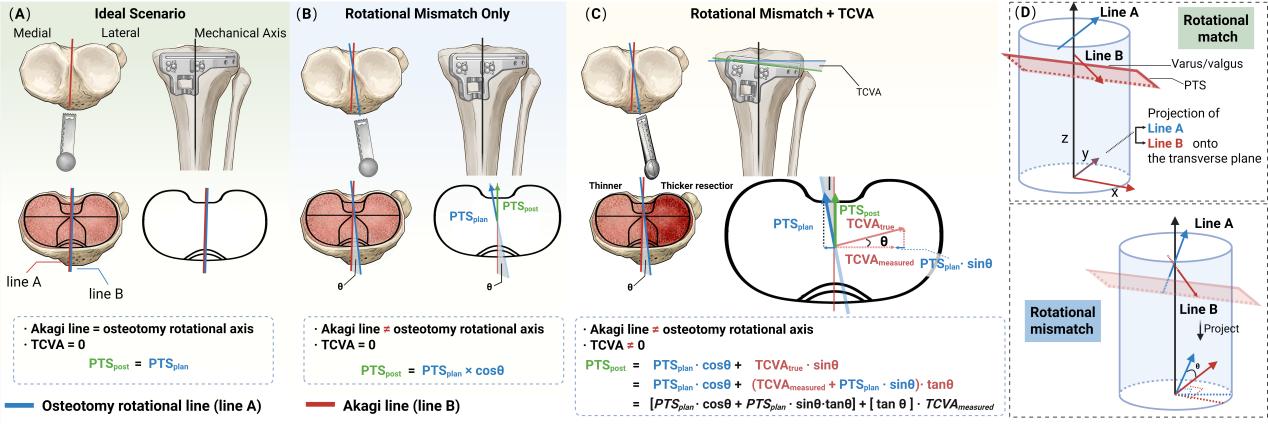
**

**Fig.** Schematic illustration of the geometric mechanism underlying iatrogenic coronal-sagittal coupling in tibial osteotomy. **(A) Ideal Scenario**: The osteotomy rotational axis (Line A) coincides with the Akagi line (Line B), and the cutting guide is aligned perpendicular to the mechanical axis (TCVA = 0°). In this case, the postoperative PTS equals the planned PTS (PTS_post = PTS_plan). **(B) Rotational Mismatch Only:** When the osteotomy rotational axis diverges from the Akagi line by an angle θ, but the cutting guide remains perpendicular to the mechanical axis (TCVA_true = 0°), only a minimal projection error occurs (PTS_post ≈ PTS_plan × cosθ). Importantly, this scenario does not produce a correlation between coronal adjustment and PTS change. **(C) Rotational Mismatch Combined with Cutting Guide Tilt:** When the extramedullary guide fails to align parallel with the tibial mechanical axis, the cutting guide tilts in the coronal plane (TCVA_true ≠0°). This coronal deviation is projected onto the sagittal plane through the θ°rotational mismatch, causing PTS_post to be simultaneously influenced by both the PTS_plan component and the TCVA_true component. (Note: TCVA_true = TCVA_measured + PTS_plan·sinθ, as the planned posterior slope also contributes a component to the measured varus/valgus angle. Furthermore, the magnitude of θcan be estimated from the regression coefficient of TCVA_measured in the final equation for PTS_post.) These two factors create a cascading effect: greater preoperative deformity → increased difficulty in guide rod placement → larger TCVA → greater PTS deviation via rotational projection. **(D) Geometric Projection Model:** Since the Akagi line and the osteotomy rotational axis do not lie in the same three-dimensional plane, we projected both reference lines onto a single two-dimensional plane perpendicular to the mechanical axis. By ignoring the irrelevant Z-axis translation (depth difference) and focusing solely on directional orientation, we calculated the “equivalent rotational mismatch” that determines the sagittal outcome. Abbreviations: PTS_post, posterior tibial slope measured on postoperative lateral radiograph; PTS_plan, planned posterior tibial slope set on the cutting guide; TCVA_true, true varus/valgus angle of the tibial cutting guide relative to the tibial mechanical axis; TCVA_measured, measured cutting guide varus/valgus angle, calculated as 90°minus postoperative MPTA.
