# Supplementary material for: Iatrogenic coronal-sagittal coupling driven by a 12.4° rotational mismatch in manual total knee arthroplasty and precise decoupling with robotic assistance: a radiographic retrospective cohort study
Source: Arthroplasty. 2026 Jun 3;8:41. doi: 10.1186/s42836-026-00398-3 (PMC13231751; doi:10.1186/s42836-026-00398-3)

**Measurement Method for Distal Femoral Flexion**

The core principle of this method is to use the anatomical axis of the distal femur to identify the most distal point of the femur. The tangent at this point is considered the distal femoral joint surface. The angle between the perpendicular line to the anatomical axis and the distal femoral joint surface is defined as the distal femoral flexion.

**STEP 1**: Import the sagittal knee joint image into the RadiAnt DICOM Viewer. Draw circles 5 cm and 10 cm away from the distal femur along the femoral shaft, and connect the circle centers to form the anatomical axis of the distal femur (green line in the image). Draw the perpendicular line to the anatomical axis (yellow line), then translate the perpendicular line distally along the femur (yellow dashed line). When the perpendicular line intersects the contour of the distal femur at exactly one point, record this point as the "most distal point." Save the image.


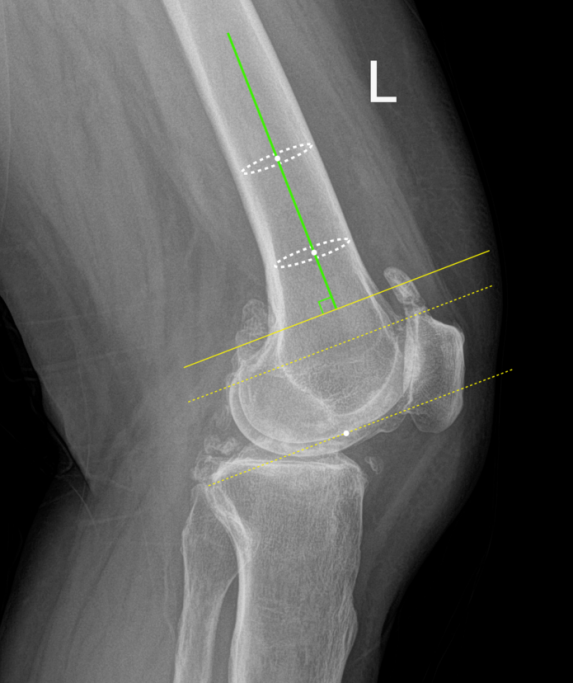
**STEP 2:** Import the image into ImageJ software. Adjust the contrast and resize the image to an appropriate dimension.

**STEP 3:** Open the Kappa plugin in ImageJ software, activate it, and select 30–40 points along the contour of the distal femur (green points in the image). Click "Fit Curve" to perform curve fitting (light blue line in the image below). Then, click "Show Tangent and Normal Vectors" in the View menu to display the tangent and normal vectors.[The attached video demonstrates this process]


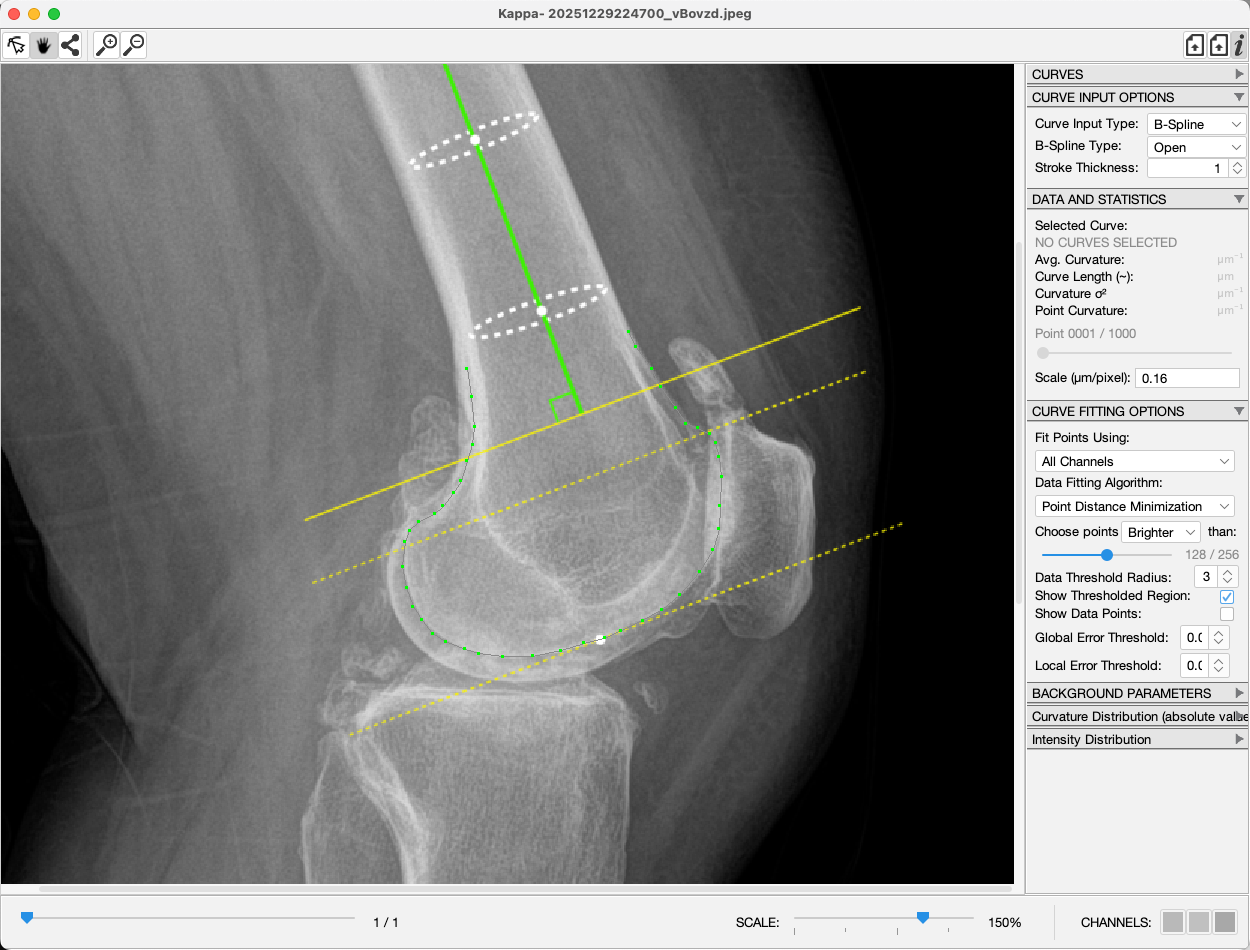

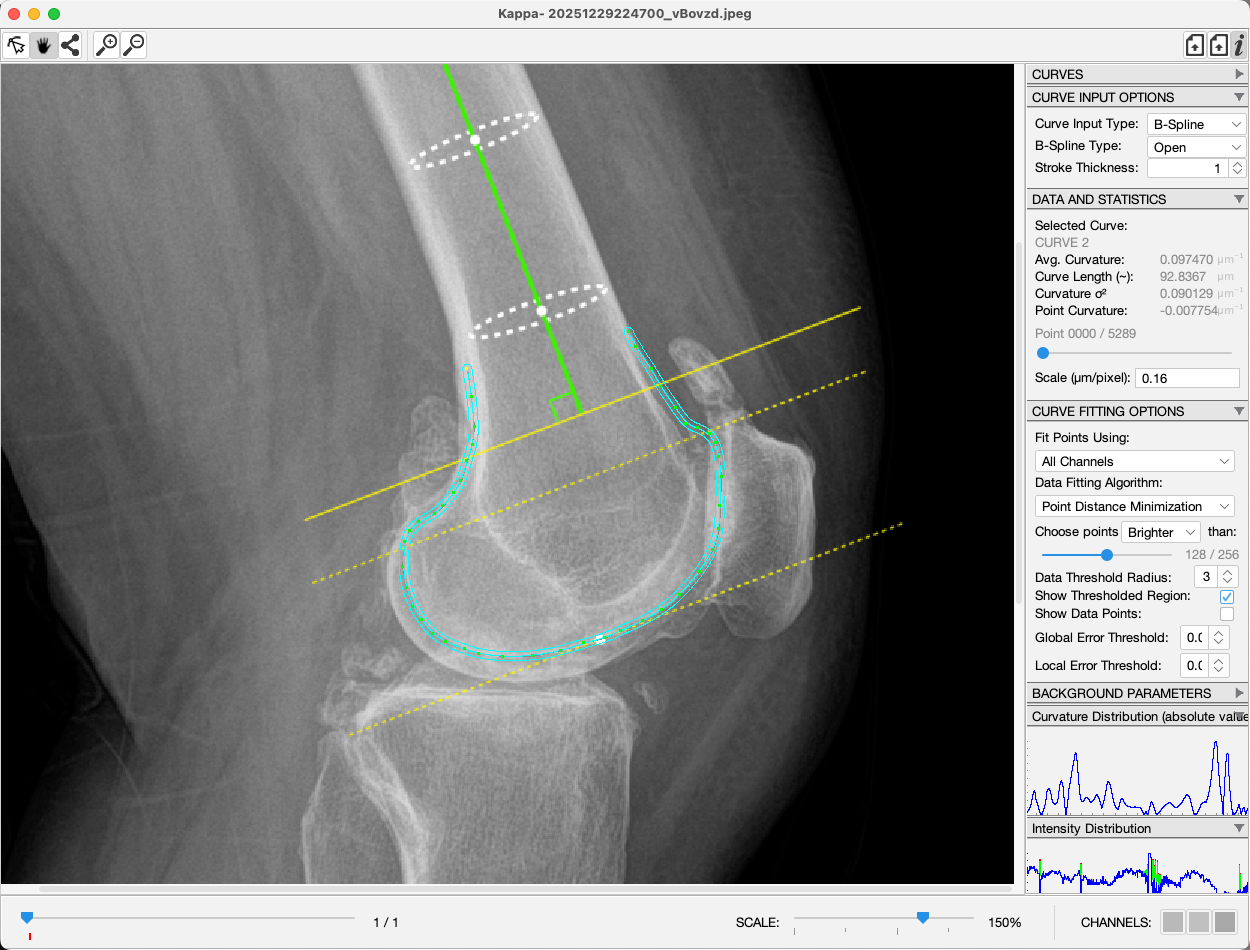


**STEP 4:**Make sure to drag the tangent point to the position of the previously marked “most distal point”.


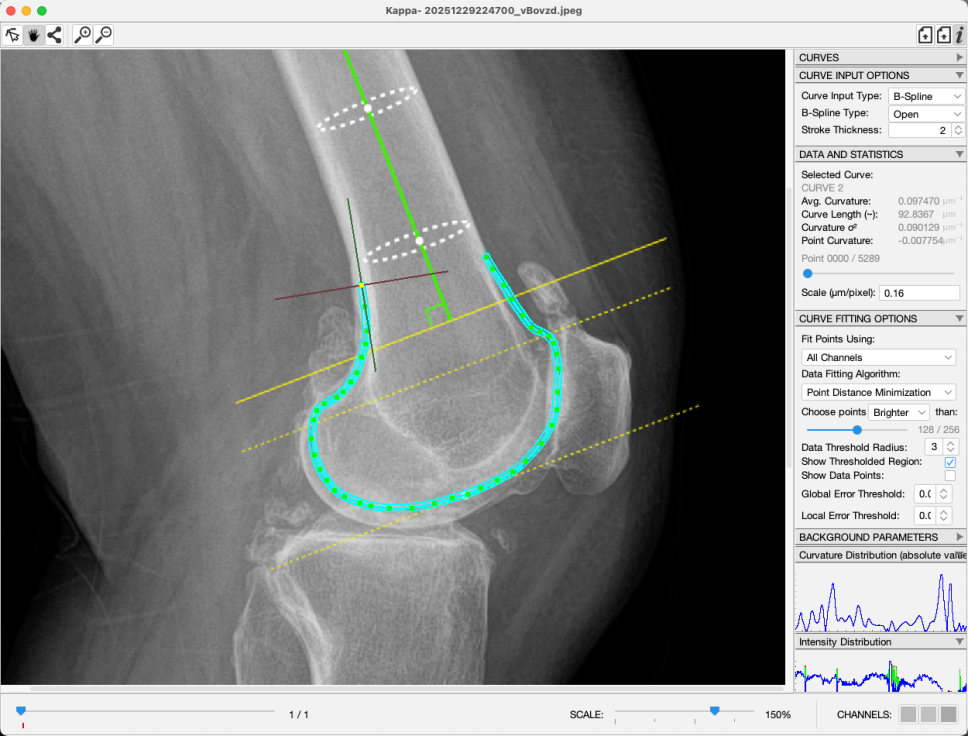

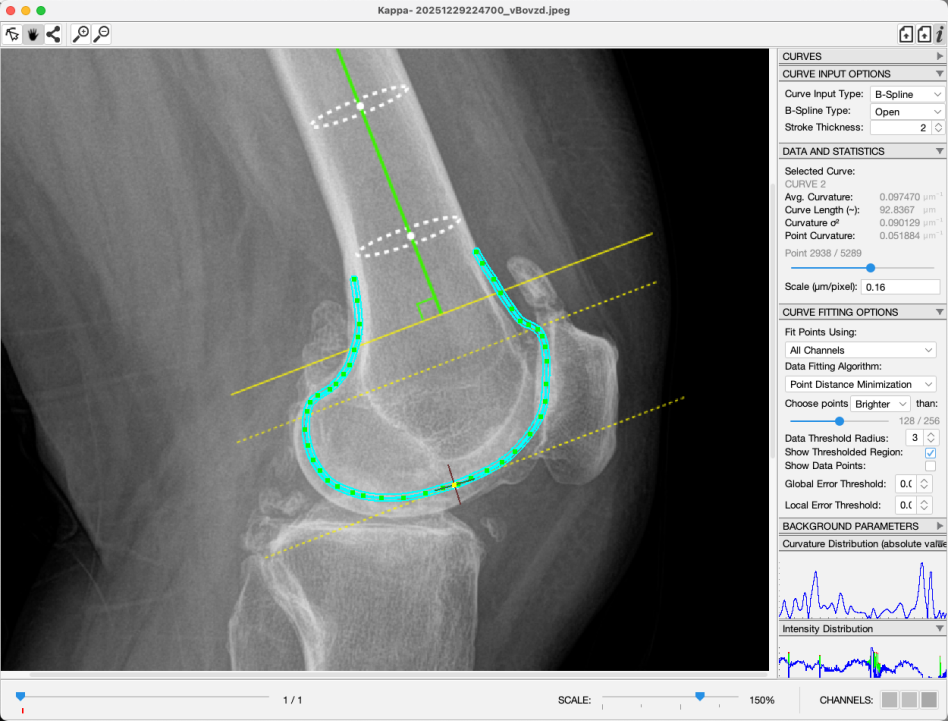


**STEP 5:**Activate the pre-written program in Matlab, open the image, and select the “tangent at the most distal point (blue)” and the “perpendicular line to the anatomical axis (yellow line)”. The system will automatically calculate the angle as 4.42 degrees.


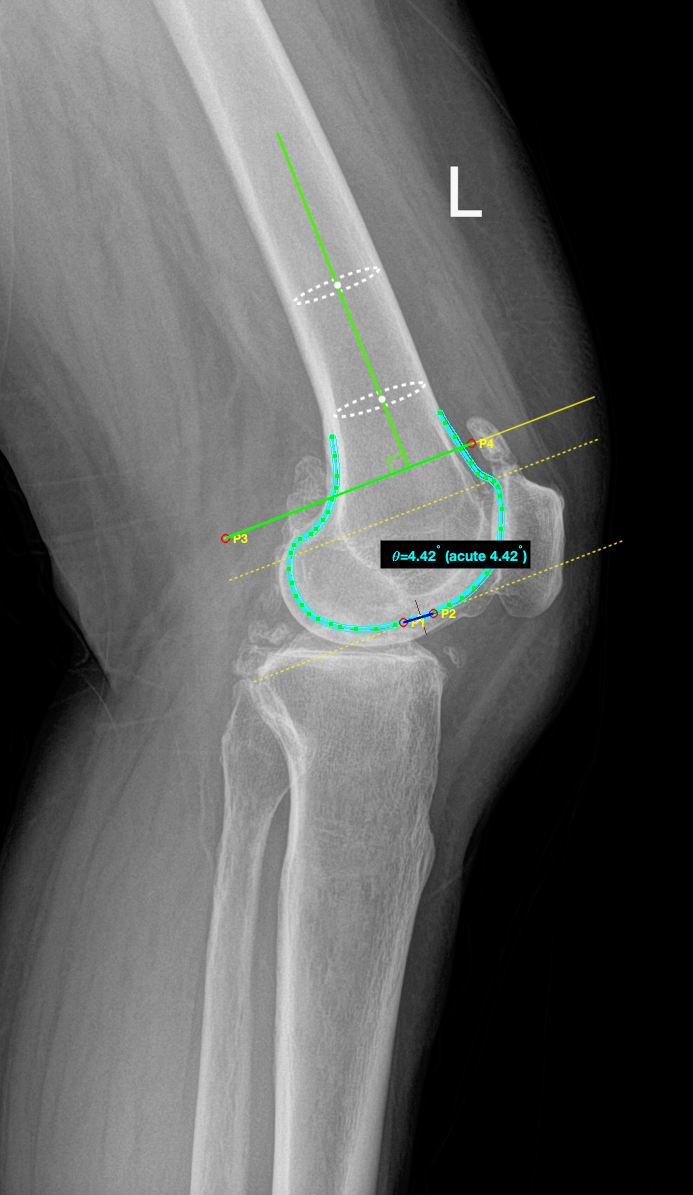

Supplement: Supplementary file 1 — Supplementary Material 1 (Measurement Method for Distal Femoral Flexion). Supplementary Material 2 (Detailed Explanation of Figure 6). Supplementary Material 3 (Detailed mechanism of the mismatch between the osteotomy axis and the tibial component placement axis in manual TKA). Supplementary Material 4 (Analysis of the plausibility of a 12.4° angle between the osteotomy rotational axis and the Akagi line). Supplementary Material 5 (Demonstration of Robotic Decoupling) and Supplementary tables (Tables S1-S4). [file 42836_2026_398_MOESM1_ESM.zip › supplementary material/supplementary material 1.Measurement Method for Distal Femoral Flexion.docx]
