# Supplementary material for: Iatrogenic coronal-sagittal coupling driven by a 12.4° rotational mismatch in manual total knee arthroplasty and precise decoupling with robotic assistance: a radiographic retrospective cohort study
Source: Arthroplasty. 2026 Jun 3;8:41. doi: 10.1186/s42836-026-00398-3 (PMC13231751; doi:10.1186/s42836-026-00398-3)
Supplement: Supplementary file 1 — Supplementary Material 1 (Measurement Method for Distal Femoral Flexion). Supplementary Material 2 (Detailed Explanation of Figure 6). Supplementary Material 3 (Detailed mechanism of the mismatch between the osteotomy axis and the tibial component placement axis in manual TKA). Supplementary Material 4 (Analysis of the plausibility of a 12.4° angle between the osteotomy rotational axis and the Akagi line). Supplementary Material 5 (Demonstration of Robotic Decoupling) and Supplementary tables (Tables S1-S4). [file 42836_2026_398_MOESM1_ESM.zip › supplementary material/supplementary material 5.Demonstration of Robotic Decoupling.docx]

**Demonstration of Robotic Decoupling**

The generation of the iatrogenic coronal–sagittal coupling effect in manual TKA requires the simultaneous presence of two necessary conditions: (1) **TCVA ≠ 0** (coronal tilt of the tibial cutting guide); and (2) a **rotational mismatch** between the osteotomy rotational reference axis and the prosthesis installation reference axis (Akagi line). Neither condition alone is sufficient—TCVA without rotational mismatch produces only a pure coronal error, whereas rotational mismatch with TCVA = 0 yields a negligible sagittal deviation (cos 12.4° ≈ 0.98, i.e. only ≈2% error).

The Mako robotic system eliminates **both** of these conditions through its integrated workflow, as illustrated step by step below.

**Step 1. Establishment of a unified three-dimensional reference coordinate system.**

Preoperatively, the engineering team reconstructs a patient-specific three-dimensional model of the lower extremity within the Mako system using CT data. In this digital model, the coronal, sagittal, and axial planes of the tibia are truly orthogonal and mutually independent in the strict mathematical sense (Figure S1).


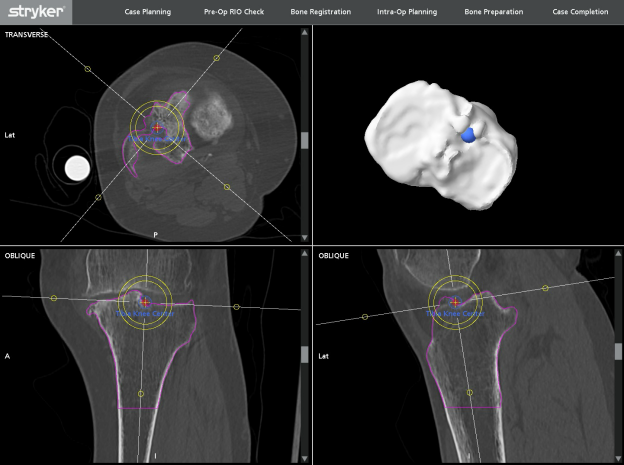

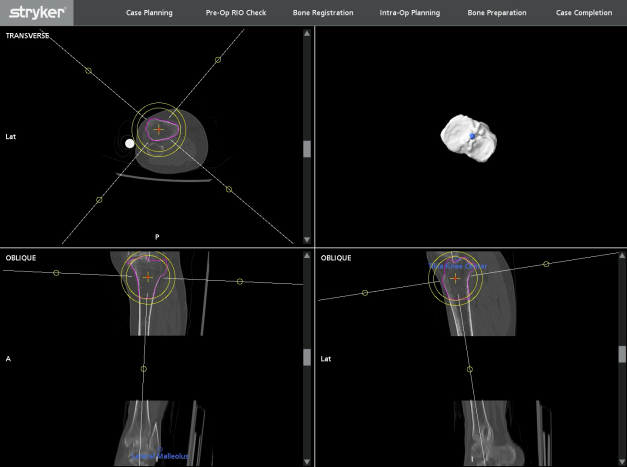


**Figure S1. Unified three-dimensional reference coordinate system generated by the Mako robotic system.** The transverse, coronal, and sagittal planes of the tibia are shown and are mutually orthogonal.

Critically, the tibial rotational reference axis is set as the **Akagi line**—defined as the line connecting the midpoint of the posterior cruciate ligament (PCL) tibial insertion to the medial border of the patellar tendon attachment. This convention has been consistently adopted in multiple studies using the Mako platform: Clark et al. explicitly stated that in 650 Mako functional-alignment TKAs, tibial rotation was “set to the Akagi line”; Yamamoto et al. defined the Akagi line as the tibial rotational reference in their Mako R-TKA study; and Giovanoulis et al., in a systematic review, concluded that among all included Mako-based studies, tibial rotation was “overwhelmingly referenced to the Akagi line.” By adopting the Akagi line as the default rotational reference during preoperative CT-based planning, the system ensures consistency between the osteotomy reference axis and the prosthesis installation reference axis from the outset—thereby **fundamentally eliminating Condition 2 (the rotational mismatch)** (Figure S2).


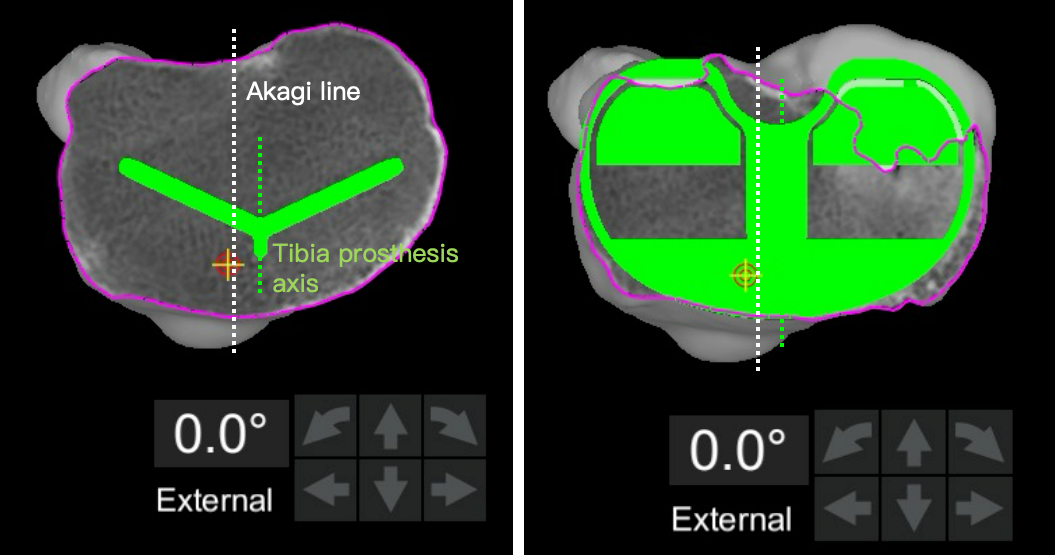


**Figure S2. Unified rotational reference based on the Akagi line.** Axial view of the proximal tibia in the Mako planning interface. The white dotted line represents the Akagi line (PCL midpoint to medial border of the patellar tendon insertion), which is adopted as the rotational reference axis for both the tibial osteotomy and the tibial component installation. The green dotted line represents the tibial prosthesis axis. Left panel: initial rotational alignment setting (0.0° external). Right panel: tibial component overlaid on the resected tibial surface, confirming that the prosthesis axis remains coincident with the planning axis (Akagi line). Because both the cutting plane and the prosthesis are referenced to the same Akagi line, the rotational mismatch inherent to manual extramedullary alignment is eliminated at the planning stage.

**Step 2. Tibial osteotomy execution (performed according to the planned parameters, without a physical cutting guide).**

Unlike manual TKA, which relies on an extramedullary guide rod (proximally aimed at the medial one-third of the tibial tubercle and distally at the ankle center) to establish the osteotomy rotational axis, the Mako system **does not use any physical cutting guide**. As established in Step 1, a unified three-dimensional coordinate system has already been built on the basis of the Akagi line. All osteotomy parameters—including the magnitude and direction of the varus/valgus angle and the posterior tibial slope (PTS)—are defined independently within this coordinate system. The “posterior” direction of the PTS now corresponds to the sagittal plane defined with the Akagi line as the rotational axis, rather than the sagittal plane defined by the extramedullary guide rod, thereby avoiding any cross-plane projection error caused by a rotational offset of the reference frame. Intraoperatively, the surgeon operates the saw attached to the robotic arm within a stereotactic haptic boundary strictly derived from the preoperative plan; any excursion beyond the pre-defined three-dimensional envelope is physically prevented by the robotic arm (Figure S3). Because no extramedullary guide rod is used, the mechanism that generates TCVA is **structurally absent**—Condition 1 is thereby completely eliminated.


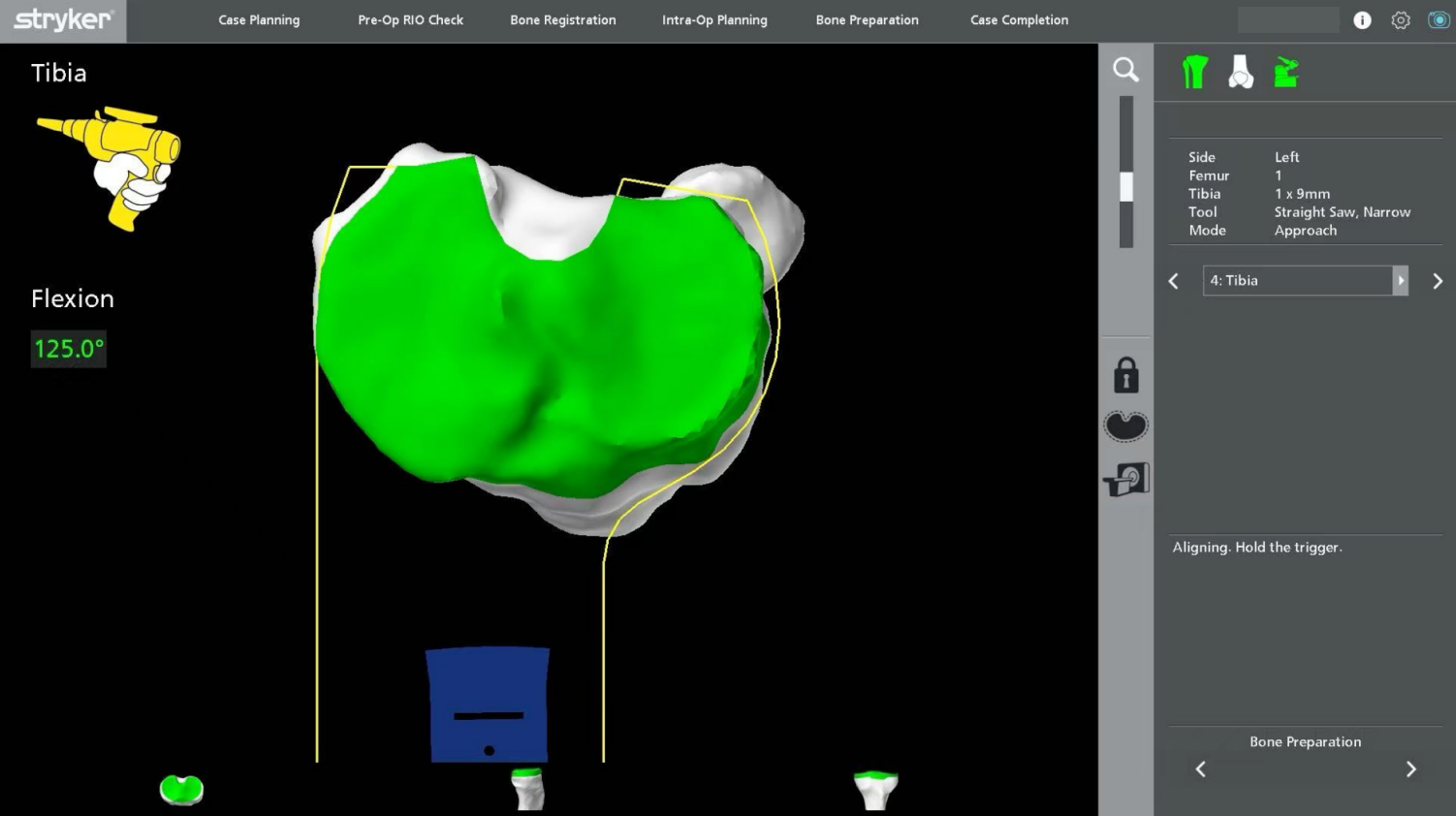


**Figure S3. Robotic-arm-assisted tibial bone preparation within a stereotactic haptic boundary.** Intraoperative screenshot of the Mako system during tibial resection. The green region indicates the pre-planned resection volume, while the yellow contour delineates the three-dimensional haptic boundary. The saw blade, mounted on the robotic arm, is physically constrained within this boundary—any attempted excursion beyond the planned envelope is mechanically prevented by the robotic arm. Because no physical extramedullary cutting guide is used, the structural mechanism that generates TCVA in manual TKA is completely absent.

**Step 3. Tibial component installation (referenced to the Akagi line, with robotic-assisted intraoperative verification).**

After completion of the osteotomy, the tibial trial is also rotationally aligned to the Akagi line. Importantly, the surgeon can verify rotational alignment intraoperatively using the robotic system: an optical probe is placed on anatomical landmarks on the bone surface (the PCL midpoint and the medial border of the patellar tendon), and their positions are confirmed against the CT-based plan of the Akagi line on the Mako display (Figure S4). This ensures that the resected surface and the final prosthesis share **one and the same rotational reference—the Akagi line**—with no angular offset between them.


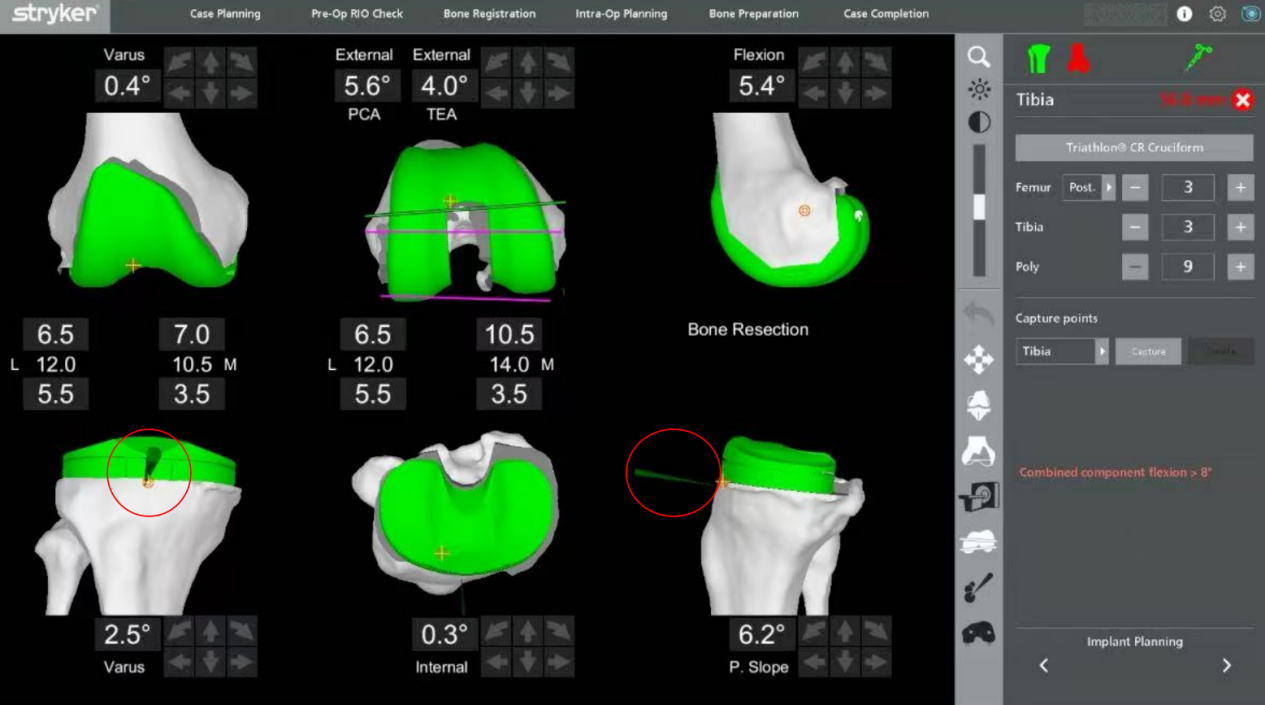


**Figure S4. Intraoperative verification of final component position against the preoperative plan.** Numerical values of varus/valgus, flexion, rotation, and posterior tibial slope are displayed for both components, allowing the surgeon to confirm that the executed alignment matches the preoperative three-dimensional plan. The red circles highlight the intraoperative verification of the anterior starting point of the Akagi line: the optical probe is placed on the visually estimated landmark on the resected tibial surface, and its position is cross-checked on the Mako display to confirm correspondence with the medial one-third of the tibial tubercle. This robotic-assisted verification provides an objective confirmation of an anatomical landmark that is otherwise identified by visual inspection alone.

**Summary.**

The robotic system achieves complete coronal–sagittal decoupling through the following mechanisms: (a) a unified CT-based three-dimensional coordinate system in which each planar parameter is orthogonally and independently defined; (b) a guide-less, haptic-bounded osteotomy that structurally eliminates TCVA; and (c) a single, consistent rotational reference (the Akagi line) maintained throughout osteotomy and component installation, thereby eliminating the ≈12.4° rotational mismatch that is inherent to the extramedullary guide system of manual TKA. As both necessary conditions for the coupling effect are removed, the iatrogenic coronal–sagittal coupling is prevented at its geometric origin.
