# Supplementary material for: Iatrogenic coronal-sagittal coupling driven by a 12.4° rotational mismatch in manual total knee arthroplasty and precise decoupling with robotic assistance: a radiographic retrospective cohort study
Source: Arthroplasty. 2026 Jun 3;8:41. doi: 10.1186/s42836-026-00398-3 (PMC13231751; doi:10.1186/s42836-026-00398-3)
Supplement: Supplementary file 1 — Supplementary Material 1 (Measurement Method for Distal Femoral Flexion). Supplementary Material 2 (Detailed Explanation of Figure 6). Supplementary Material 3 (Detailed mechanism of the mismatch between the osteotomy axis and the tibial component placement axis in manual TKA). Supplementary Material 4 (Analysis of the plausibility of a 12.4° angle between the osteotomy rotational axis and the Akagi line). Supplementary Material 5 (Demonstration of Robotic Decoupling) and Supplementary tables (Tables S1-S4). [file 42836_2026_398_MOESM1_ESM.zip › supplementary material/supplementary material 3.Detailed mechanism of the mismatch between the ost.docx]

**Detailed mechanism of the mismatch between the osteotomy axis and the tibial component placement axis in manual TKA**

**1. Geometric Validation Using a Patient-Specific Three-Dimensional Model**

We randomly selected one patient from our study cohort and processed the preoperative CT data to reconstruct a three-dimensional anatomical model of the right tibia using Blender software (v4.0, Blender Foundation, Amsterdam, Netherlands). On this reconstructed model, we annotated the following key anatomical landmarks:

1. **Medial one-third of the tibial tubercle** (red marker): the ideal placement point for the proximal reference of the extramedullary alignment rod, which also serves as the anterior defining point of the Akagi line [1,2];
2. **3–5 mm medial to the ankle center** (red marker): the ideal placement point for the center of the ankle clamp. According to Siston et al. [3] and a recent deep-learning–based radiological analysis [4], the true mechanical center of the ankle lies approximately 3–5 mm medial to the midpoint of the intermalleolar line; consequently, the ankle clamp is clinically positioned with this offset to more closely approximate the true tibial mechanical axis;
3. **Medial border of the posterior cruciate ligament** (blue marker): the posterior defining point of the Akagi line [1].

We then projected the Akagi line (red solid line, connecting the medial PCL border and the medial one-third of the tibial tubercle) onto the axial plane of the tibial plateau. Following the specifications of the Stryker Triathlon MIS surgical technique manual, the modeled extramedullary alignment rod assembly was precisely positioned on the tibia — with its proximal reference arm aligned to the medial one-third of the tibial tubercle and its distal ankle clamp centered 3–5 mm medial to the ankle center (**Figure S1**).


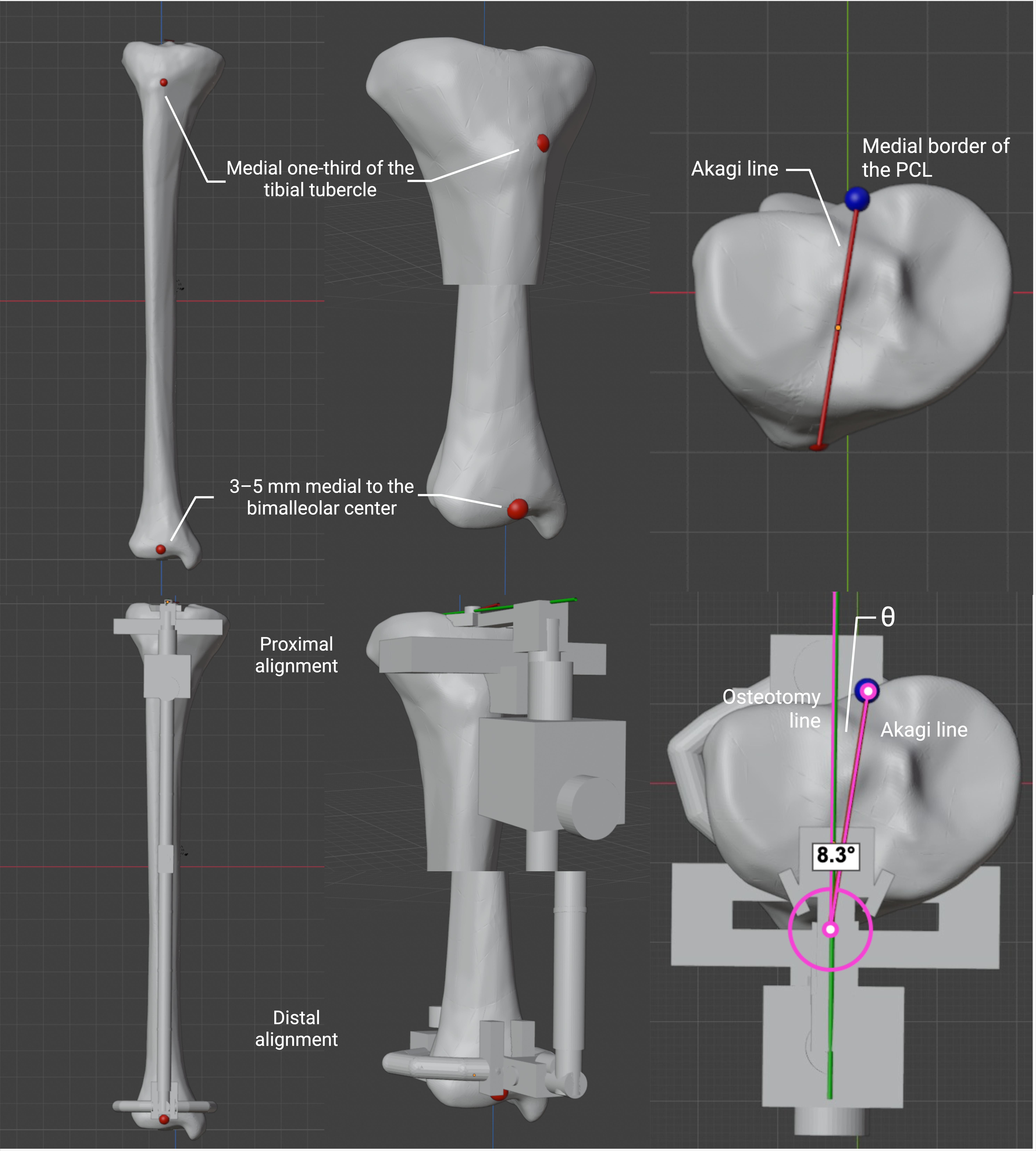


**Figure S1 Geometric mechanism of the angular mismatch between the osteotomy axis and the Akagi line in manual TKA.** Upper panel: annotation of the relevant tibial anatomical landmarks. Lower panel: even when the extramedullary alignment rod is placed precisely according to these anatomical landmarks, the osteotomy axis and the Akagi line remain non-coincident.

Under these ideal placement conditions, the angle measured between the rotational reference axis of the cutting guide and the Akagi line on the axial projection was **8.3°**. This finding directly validates our hypothesis: even when the extramedullary alignment rod is positioned strictly according to anatomical landmarks, an irreducible systematic angular mismatch Δθ persists between the two axes.

**2. Supporting Evidence from the Literature**

This angular discrepancy is not an incidental phenomenon; rather, it represents a systematic error arising from the fact that the anatomical landmarks defining the two reference frames are not collinear in three-dimensional space:

- The posterior reference point of the Akagi line (medial PCL border) lies slightly medial to the midline of the tibial plateau, whereas the distal reference point of the extramedullary alignment rod (medial to the ankle center) is located at the distal tibia and is subject to axial offset due to tibial torsion. A CT-based study by Graw et al. [5] demonstrated that tibial torsion induces substantial rotational deviation of the extramedullary guide's distal reference point relative to its proximal reference.
- An MRI-based three-dimensional simulation by Tsukeoka et al. [6] showed that, even when the bony malleolar prominences are accurately identified, the mean lateral error between the intermalleolar center and the true talar center remains 3.7 ± 1.4 mm.
- Furthermore, an MRI study [7] reported that, in the general population, the axis passing through the medial one-third of the tibial tubercle is externally rotated by a mean of 0.5° ± 4.4° relative to the femoral transepicondylar axis (TEA), whereas the Akagi line is internally rotated by 3.7° ± 4.5°. The two axes are thus inherently non-coincident even in healthy individuals, and anatomical variations in osteoarthritic patients are expected to further amplify this discrepancy.

**3. Clinical Consequences of the Angular Mismatch**

When an angular mismatch Δθ exists between the osteotomy axis and the Akagi line, if the surgeon sets α degrees of posterior slope (e.g., the 3° posterior slope commonly used for CR prostheses) and performs the osteotomy along the instrument axis but ultimately seats the prosthesis along the Akagi line, the postoperatively measured posterior tibial slope (PTS) is, in fact, the projection of the originally set slope onto the Akagi line (as illustrated in the original manuscript, **Figure 6(B), Rotational Mismatch Only**):

- True posterior slope = α · cos(Δθ)
- Coronal coupling component (varus/valgus) = α · sin(Δθ)

When the prosthesis is finally aligned along the Akagi line, the coronal coupling component manifests as an “unintended” varus/valgus deviation — which is precisely the geometric origin of the coronal-sagittal coupling reported in our study.

It should be emphasized that this demonstration was performed on an anatomically near-normal tibial model. In actual clinical practice, the real Δθ is typically larger due to soft-tissue obscuration of bony landmarks, proximal tibial osteophytes, variability in tibial torsion, and restricted intraoperative visualization [8,9]. The mean coupling angle of approximately 12° reported in our study is therefore consistent with — and biologically plausible within — a real-world surgical environment.

**References**

[1] Akagi M, Oh M, Nonaka T, Tsujimoto H, Asano T, Hamanishi C. An anteroposterior axis of the tibia for total knee arthroplasty. *Clin Orthop Relat Res*. 2004;(420):213–219.

[2] Lavernia C, Contreras JS, Alcerro JC. Rotational alignment of the tibial component in total knee arthroplasty is better at the medial third of the tibial tuberosity than at the medial border. *Clin Orthop Relat Res*. 2010;468(4):1007–1014.

[3] Siston RA, Daub AC, Giori NJ, Goodman SB, Delp SL. Evaluation of methods that locate the center of the ankle for computer-assisted total knee arthroplasty. *Clin Orthop Relat Res*. 2005;439:129–135.

[4] Comparison of tibial alignment parameters based on clinically relevant anatomical landmarks: a deep learning radiological analysis. *Bone Jt Open*. 2022;3(10):767–776.

[5] Graw BP, Harris AH, Tripuraneni KR, Giori NJ. Effects of tibial torsion on distal alignment of extramedullary instrumentation in total knee arthroplasty. *Acta Orthop*. 2010;81(5):615–619.

[6] Tsukeoka T, Tsuneizumi Y, Lee TH. Estimation of frontal alignment error of the extramedullary tibial guide on the bi-malleolar technique: a simulation study with magnetic resonance imaging. *Knee*. 2012;19(5):634–638.

[7] Kim YT, Kang MW, Lee JK, Lee YM, Kim JI. Evaluation of tibial rotational axis in total knee arthroplasty using magnetic resonance imaging. *Sci Rep*. 2020;10(1):14068.

[8] Reed MR, Bliss W, Sher JL, Emmerson KP, Jones SM, Partington PF. Extramedullary or intramedullary tibial alignment guides: a randomised, prospective trial of radiological alignment. *J Bone Joint Surg Br*. 2002;84(6):858–860.

[9] Rivière C, Iranpour F, Auvinet E, Howell S, Vendittoli PA, Cobb J, Parratte S. Alignment options for total knee arthroplasty: a systematic review. *Orthop Traumatol Surg Res*. 2017;103(7):1047–1056.
