# Supplementary material for: Iatrogenic coronal-sagittal coupling driven by a 12.4° rotational mismatch in manual total knee arthroplasty and precise decoupling with robotic assistance: a radiographic retrospective cohort study
Source: Arthroplasty. 2026 Jun 3;8:41. doi: 10.1186/s42836-026-00398-3 (PMC13231751; doi:10.1186/s42836-026-00398-3)
Supplement: Supplementary file 1 — Supplementary Material 1 (Measurement Method for Distal Femoral Flexion). Supplementary Material 2 (Detailed Explanation of Figure 6). Supplementary Material 3 (Detailed mechanism of the mismatch between the osteotomy axis and the tibial component placement axis in manual TKA). Supplementary Material 4 (Analysis of the plausibility of a 12.4° angle between the osteotomy rotational axis and the Akagi line). Supplementary Material 5 (Demonstration of Robotic Decoupling) and Supplementary tables (Tables S1-S4). [file 42836_2026_398_MOESM1_ESM.zip › supplementary material/supplementary material 4.Analysis of the plausibility of a 12.4┬░ angle between the osteotomy rotational axis and the Akagi line.docx]

**Analysis of the plausibility of a 12.4° angle between the osteotomy rotational axis and the Akagi line**

1. Insall line, originally proposed by John Insall, is a two-dimensional reference line based on anatomical landmarks on the proximal tibial resection surface. Proximal landmark: the center of the tibial insertion of the posterior cruciate ligament (PCL). Distal landmark: the medial one-third of the tibial tubercle. [1]
2. The Akagi line[2]was described in detail by Akagi et al. in 2004 to identify a tibial reference line perpendicular to the femoral surgical transepicondylar axis (sTEA), thereby achieving rotational matching of the flexion and extension gaps. Proximal landmark: identical to the Insall line. Distal landmark: the medial border of the patellar tendon insertion, which typically corresponds to the medial border of the tibial tubercle.
3. Operational axis of the extramedullary alignment rod: proximally referenced to the medial one-third of the tibial tubercle; distally referenced to the medial aspect of the ankle center, aligned toward the axis of the second metatarsal.

Lu[3]et al. reported that the Insall line is externally rotated by 11.9°relative to the sTEA, whereas the Akagi line is externally rotated by 1.4°relative to the sTEA, which is broadly consistent with previously published results[4, 5]. The difference between these values allows us to infer that the Insall line is externally rotated by approximately 10.5° relative to the Akagi line. As no prior study has directly measured the axial-plane angle between the “proximal medial one-third tibial tubercle–distal second metatarsal line” and the Akagi line, we performed a geometric derivation using available anatomical data to validate the plausibility of the 12.4° angle observed in our study. This angle is determined by two components:

1. Difference in proximal landmarks: medial one-third of the tibial tubercle vs. medial border of the tibial tubercle.
2. Alignment of distal landmarks: second metatarsal axis vs. the distal projection of the Akagi line.

Anatomically, the “medial one-third” of the tibial tubercle lies lateral to its “medial border.” Using the right knee as an example, a ray originating from the PCL center and directed toward the medial one-third (Insall line) will necessarily be oriented more laterally—i.e., more externally rotated—than a ray directed toward the medial border (Akagi line).

The existing literature is highly consistent in indicating that, considering the proximal landmark alone, an axis referencing the **medial one-third of the tibial tubercle** is approximately **9° to 10.5°** more externally rotated than an axis referencing the **medial border** (Akagi line).

**Effect of the distal landmark (second metatarsal vs. mechanical axis):** In standard anatomical models, the second metatarsal axis is commonly considered to coincide with the sagittal-plane projection of the tibial mechanical axis, and thus to be broadly parallel to the distal extension of the Akagi line. In a recent 3D-CT study, Uehara et al.[6]compared the Akagi line with the second metatarsal axis and reported a mean deviation of **−0.85° ± 11.18°** for the second metatarsal axis relative to the Akagi line. This suggests that, on average, the second metatarsal axis is nearly parallel to the Akagi line (with a mean deviation of <1°).

**Inference:** If the distal reference (second metatarsal) is nearly parallel to the Akagi line (≈0° deviation), while the proximal reference (medial one-third of the tibial tubercle) is externally rotated by ~10° relative to the Akagi line, then the overall rotational orientation of the extramedullary alignment rod (proximal medial one-third tibial tubercle + distal second metatarsal) should be predominantly driven by the proximal landmark offset. Accordingly, the extramedullary alignment axis would be expected to exhibit approximately **10° of external rotation** relative to the Akagi line.

Considering the standard deviation (SD) associated with inter-individual anatomical variability, this angle would typically span approximately **7° to 12°**, which further supports the plausibility of our observed value of **12.4°**.

**Reference:**

1. Lu, Y., et al., *A CT study of tibiofemoral rotation alignment in normal knee joint of Chinese adult*. 2020.

2. Mitsuhashi, S., et al., *Combined CT-based and image-free navigation systems in TKA reduces postoperative outliers of rotational alignment of the tibial component.* Arch Orthop Trauma Surg, 2018. **138**(2): p. 259-266.

3. Lu, Y., et al., *Tibiofemoral rotation alignment in the normal knee joints among Chinese adults: a CT analysis.* BMC Musculoskelet Disord, 2020. **21**(1): p. 323.

4. Zhang, H., et al., *Determining the rotational alignment of the tibial component referring to the tibial tubercle during total knee arthroplasty: the tibial tubercle-trochlear groove can be an aid.* J Orthop Surg Res, 2022. **17**(1): p. 253.

5. Panicker, J. and J. Thilak, *A computed tomographic evaluation of femoral and tibial rotational reference axes in total knee arthroplasty.* Sicot j, 2023. **9**: p. 4.

6. Uehara, T., et al., *3D Imaging Evaluation of Distal Reference Axes for Tibial Rotational Alignment in Medial-Type Knee Osteoarthritis.* Cureus, 2025. **17**(11): p. e97381.
